# Evolution of Antibiotic Resistance in Surrogates of *Francisella tularensis* (LVS and *Francisella novicida*): Effects on Biofilm Formation and Fitness

**DOI:** 10.3389/fmicb.2020.593542

**Published:** 2020-10-30

**Authors:** Fabrice V. Biot, Beth A. Bachert, Kevin D. Mlynek, Ronald G. Toothman, Galina I. Koroleva, Sean P. Lovett, Christopher P. Klimko, Gustavo F. Palacios, Christopher K. Cote, Jason T. Ladner, Joel A. Bozue

**Affiliations:** ^1^Institut de Recherche Biomédicale des Armées, Département de Biologie des Agents Transmissibles, Unité de Bactériologie/UMR_MD1, Brétigny-sur-Orge, France; ^2^Bacteriology Division, U.S. Army Medical Research Institute of Infectious Diseases (USAMRIID), Frederick, MD, United States; ^3^Center for Genome Sciences, U.S. Army Medical Research Institute of Infectious Diseases (USAMRIID), Frederick, MD, United States

**Keywords:** *Francisella*, *Francisella novicida*, LVS, antimicrobial resistance, streptomycin, ciprofloxacin, tularemia, biofilm

## Abstract

*Francisella tularensis*, the causative agent of tularemia, is capable of causing disease in a multitude of mammals and remains a formidable human pathogen due to a high morbidity, low infectious dose, lack of a FDA approved vaccine, and ease of aerosolization. For these reasons, there is concern over the use of *F. tularensis* as a biological weapon, and, therefore, it has been classified as a Tier 1 select agent. Fluoroquinolones and aminoglycosides often serve as the first line of defense for treatment of tularemia. However, high levels of resistance to these antibiotics has been observed in gram-negative bacteria in recent years, and naturally derived resistant *Francisella* strains have been described in the literature. The acquisition of antibiotic resistance, either natural or engineered, presents a challenge for the development of medical countermeasures. In this study, we generated a surrogate panel of antibiotic resistant *F. novicida* and Live Vaccine Strain (LVS) by selection in the presence of antibiotics and characterized their growth, biofilm capacity, and fitness. These experiments were carried out in an effort to (1) assess the fitness of resistant strains; and (2) identify new targets to investigate for the development of vaccines or therapeutics. All strains exhibited a high level of resistance to either ciprofloxacin or streptomycin, a fluoroquinolone and aminoglycoside, respectively. Whole genome sequencing of this panel revealed both on-pathway and off-pathway mutations, with more mutations arising in LVS. For *F. novicida*, we observed decreased biofilm formation for all ciprofloxacin resistant strains compared to wild-type, while streptomycin resistant isolates were unaffected in biofilm capacity. The fitness of representative antibiotic resistant strains was assessed *in vitro* in murine macrophage-like cell lines, and also *in vivo* in a murine model of pneumonic infection. These experiments revealed that mutations obtained by these methods led to nearly all ciprofloxacin resistant *Francisella* strains tested being completely attenuated while mild attenuation was observed in streptomycin resistant strains. This study is one of the few to examine the link between acquired antibiotic resistance and fitness in *Francisella* spp., as well as enable the discovery of new targets for medical countermeasure development.

## Introduction

The facultative intracellular gram-negative bacterium *Francisella tularensis*, found ubiquitously across the northern hemisphere, is responsible for the zoonotic disease known as tularemia or more commonly “rabbit fever.” The disease is typically spread between hosts by arthropod vectors, namely through the bite of an infected mosquito or tick (Ellis et al., 2002). *F. tularensis* has a broad host range, as infections have been noted in an array of vertebrates including amphibians, birds, and mammals, though preferential colonization has been observed in lagomorphs in North America, mainly hares, and rabbits (Jellison and Parker, 1945; Hopla, 1974; Morner, 1992). The ulceroglandular form of tularemia is the most common, resulting in a primary ulcer at the inoculation site, followed by regional lymphadenopathy. Less common disease outcomes, depending on the route of exposure, include ocular, oropharyngeal, typhoidal, and pneumonic forms, with pneumonic tularemia having higher mortality rates (Hepburn and Simpson, 2008).

*F. tularensis* is comprised of two clinically important subspecies, subsp. *tularensis* (Type A) and subsp. *holarctica* (Type B), which are responsible for the majority of tularemia infections in humans. Type A strains are prevalent in North America and highly virulent, while Type B strains are present more widely across the Northern hemisphere and are typically less virulent. A third closely related subspecies, *Francisella novicida*, is only associated with brackish water and soil, and rarely causes disease in humans. *F. novicida* is commonly used as a laboratory surrogate since it has a high degree of genetic similarity to the virulent *F. tularensis* subspecies, is able to infect macrophages *in vitro* and cause disease in mice, and can be handled under BSL-2 conditions (Kingry and Petersen, 2014).

*F. tularensis* has garnered significant attention in recent years due to its concern as a potential biothreat agent. It has a low infectious dose, reported to be as low as a single bacterium, and is easy to obtain from the environment and aerosolize (Jones et al., 2005). Moreover, there is no United States (U.S.) Food and Drug Administration (FDA)-approved vaccine available, and the disease has an estimated mortality rate of 30–60% if left untreated (Pechous et al., 2009). Historically, bioweapons containing *F. tularensis* were developed during WWII by multiple biological weapons programs in the U.S., Japan, and the Soviet Union (Barras and Greub, 2014). A live vaccine strain (LVS), derived from *F. holarctica*, was developed by the Soviet Union and gifted to the U.S. and is also used as a surrogate strain. Currently, LVS is only available as an investigational vaccine to at-risk laboratory personnel (Oyston and Quarry, 2005). Moreover, its exact basis for loss of virulence is unknown, although probable genetic alterations have been identified (Rohmer et al., 2006), and concerns about its reversion to virulence and breakthrough in protection has prevented licensure by the FDA (Eigelsbach and Downs, 1961; Saslaw et al., 1961; Hornick and Eigelsbach, 1966). Complicating this threat, *F. tularensis* has the potential to acquire antibiotic resistance, either through natural selection or intentionally engineered, rendering current treatments ineffective (Loveless et al., 2010; Caspar et al., 2017; Chance et al., 2017; Heine et al., 2017). Current clinical, vector, and zoological surveillance efforts suggest emergence of resistance to clinically relevant antibiotics for *F. tularensis* strains is not typical or detected to date (Müller et al., 2013; Tomaso et al., 2017; Caspar et al., 2018). However, significant efforts are still required to i) characterize the fitness of such antibiotic resistant strains, and ii) identify novel targets for the development of vaccines and therapeutics to combat this threat.

The aminoglycosides, streptomycin and gentamicin, have been utilized historically to treat tularemia; however, issues with toxicity has kept their use mainly restricted to severe cases, while fluoroquinolones and tetracyclines are considered a first-line of defense against these infections (Hepburn and Simpson, 2008). Specifically, oral treatment with ciprofloxacin or doxycycline for at least 14 days is recommended in the event of a large-scale exposure for post-exposure prophylaxis (Dennis et al., 2001). However, relapse and treatment failure occurs in 10–15% of tularemia patients given fluoroquinolones (Johansson et al., 2001; Perez-Castrillon et al., 2001). Previous work by Sutera et al. (2014), showed that passaging of LVS and *F. novicida* on increasing concentrations of ciprofloxacin led to mutations within the topoisomerase and gyrase genes, the targets of ciprofloxacin, as well as cross-resistance to the related fluoroquinolones levofloxacin and moxifloxacin (Sutera et al., 2014). Most of the mutations were found within the QRDR (quinolone resistance-determining region) of *gyrA/B* and *parC/E*, encoding subunits of gyrase and topoisomerase enzymes, respectively. Similarly, a study focused on rapid detection of mutations leading to ciprofloxacin resistance, found that passaging of the fully virulent *F. tularensis* Schu S4 strain on ciprofloxacin gave rise to mutations in the QRDR of *gyrA* and *parE* genes (Loveless et al., 2010). Indeed, the presence of mutations in the QRDR in response to ciprofloxacin resistance is well established across diverse bacteria (Yoshida et al., 1990; Jacoby, 2005; Maruri et al., 2012). Streptomycin, typically used in severe cases of tularemia, is an aminoglycoside that inhibits bacterial protein synthesis by binding to the S12 protein of the 30S ribosomal subunit (Luzzatto et al., 1968; Parker, 2001). Like ciprofloxacin, resistance to streptomycin can occur via mutations in the *rpsL* allele encoding the ribosomal target, thereby altering the binding affinity of the drug.

The formation of a biofilm by bacterial cells is a well-known contributing factor to virulence and antibiotic resistance in many pathogenic species, often leading to recalcitrance and, subsequently, reoccurring infections (Costerton et al., 1999; Hall and Mah, 2017). Analysis of biofilm formation in recent years has revealed that *F. novicida* readily forms a robust biofilm on a variety of surfaces while the human pathogenic *F. tularensis* type A and B strains form either a weak or unsubstantial biofilm *in vitro* (Margolis et al., 2010; Mahajan et al., 2011; Champion et al., 2019). However, the importance of biofilm formation on virulence and in the treatment of tularemia is not well understood given the intracellular lifestyle of this pathogen. Previous work has shown *F. novicida* biofilms are resistant to antibiotics and that the enzymatic removal of the biofilm matrix is capable of re-sensitizing the cells embedded within the biofilm to antibiotics (Chung et al., 2014). In LVS, variation in surface polysaccharide and glycosylation has been shown to inhibit biofilm formation in wild-type cells, however, phase variation during extended incubations may remodel the cell surface to promote biofilm formation (Champion et al., 2019). While these studies have focused on the effects of biofilms on antibiotic treatment, little is currently known about how intrinsic antibiotic resistance affects biofilm formation in *Francisella*.

The emergence of multi-drug resistant bacteria has renewed the interest and urgency of understanding how the acquisition of antibiotic resistance affects virulence and alters the threat of pathogens, especially for bacterial biothreats. In this study, we selected for an antibiotic resistant panel of *Francisella* surrogates (*F. novicida* and LVS) in an effort to understand how resistance to ciprofloxacin and streptomycin affects *Francisella* fitness, which could help identify new druggable targets for medical countermeasures. Through step-wise serial passaging in the presence of these antibiotics, we show that high-level resistance to ciprofloxacin and streptomycin leads to a number of both on- and off-pathway mutations. On-pathway mutations were identified in the gyrase/topoisomerase genes and ribosomal genes, the targets of ciprofloxacin and streptomycin, respectively, as well as in genes encoding drug efflux proteins. Interestingly, many off-pathway mutations occurred in known or putative virulence factors, such as LPS and peptidoglycan biosynthesis genes. We also determined that ciprofloxacin resistant *F. novicida* strains were significantly impaired for biofilm formation compared to the parent strain, while streptomycin resistant *F. novicida* were not affected. Moreover, several ciprofloxacin resistant strains were reduced for replication in macrophages and exhibited significantly decreased virulence during intranasal infection of mice. In contrast, only mild attenuation was observed in several streptomycin resistant isolates. Here, we provide a thorough characterization of the growth, biofilm capacity, and fitness of naturally derived antibiotic resistant variants of *F. novicida* and LVS. These studies add to the growing body of knowledge on the relationship between resistance, biofilm, and virulence in bacterial species. More specifically, the mutations identified in these studies derived by serial passaging reveal genes that are involved in virulence and represent new targets to be explored for medical countermeasures to treat tularemia.

## Materials and Methods

### Bacterial Strains and Culture Conditions

Bacterial strains used in this study are listed in Table 1. *F. novicida* and LVS were routinely cultured on enriched chocolate agar plates (Thermo Fisher Scientific, Waltham, MA) at 37°C. For liquid culture, either brain heart infusion broth (BHI) supplemented with 1% IsoVitaleX (Becton-Dickinson, Cockeysville, MD) or Chamberlain’s Defined Medium (CDM) (Chamberlain, 1965).

**TABLE 1:** Antibiotics susceptibilities of CipR and StrepR variants of Fn and LVS.

CipR variants					StrepR variants		
	MIC (μg mL^–1^)					MIC (μg mL^–1^)	
Strain	CIP	DOX	CHL	PMB	Strain	STR	PMB
***Francisella novicida***							
Fn WT (U112)	0.008	0.25	0.75	96	Fn parental	8	96
Fn Cip80-1	>32	1.5	1.5	128	Fn Strep512-1	>1,024	96
Fn Cip80-2	>32	2	2	128	Fn Strep512-2	>1,024	96
Fn Cip80-3	>32	1.5	1.5	128	Fn Strep512-3	>1,024	96
Fn Cip80-4	>32	2	1.5	128	Fn Strep512-4	>1,024	96
Fn Cip80-5	>32	2	1.5	128	Fn Strep512-5	>1,024	96
Fn Cip80-6	>32	1.5	1.5	128	Fn Strep512-6	>1,024	96
Fn Cip80-7	>32	2	2	128	Fn Strep512-7	>1,024	96
Fn Cip80-8	>32	2	2	128	Fn Strep512-8	>1,024	96
Fn Cip80-9	>32	1.5	2	128	Fn Strep512-9	>1,024	96
Fn Cip80-10	>32	2	2	128	Fn Strep512-10	>1,024	96
**LVS**							
LVS WT	0.006	0.125	0.75	>1,024	LVS parental	2	>1,024
LVS Cip128-1	>32	0.75	3	256	LVS Strep512-1	>1,024	>1,024
LVS Cip128-3	>32	1	4	128	LVS Strep512-2	>1,024	>1,024
LVS Cip128-4	>32	0.75	2	384	LVS Strep512-3	>1,024	>1,024
LVS Cip128-5	>32	0.75	3	256	LVS Strep512-4	>1,024	>1,024
LVS Cip128-6	>32	1	4	128	LVS Strep512-5	>1,024	512/>1,024
LVS Cip128-7	>32	1	4	256	LVS Strep512-6	>1,024	512/>1,024
LVS Cip128-9	>32	0.75	4	128	LVS Strep512-7	>1,024	768/>1,024
LVS Cip128-10	>32	1	4	384	LVS Strep512-8	>1,024	768/>1,024
LVS Cip128-12	>32	4	24	256	LVS Strep512-9	>1,024	>1,024
					LVS Strep512-10	>1,024	>1,024

*Antibiotic sensitivities were assessed by E-tests. CIP, ciprofloxacin; DOX, doxycycline; CHL, chloramphenicol; PMB, polymyxin B; STR, streptomycin.*

### Natural Selection of Antibiotic Resistant Non-select Agent *Francisella* Strains

The surrogates *F. novicida* U112 (Larson et al., 1955) and LVS (Saslaw et al., 1961) were obtained from the USAMRIID bacterial repository and used as the parental strains to derive either ciprofloxacin or streptomycin resistant isolates. Ciprofloxacin resistant derivative strains were obtained by sequentially passaging with the sub-inhibitory concentrations of 0.0125, 0.025, 0.05, 0.1, 0.2, 0.5, 1, 2, 4, 8, 16, 32, 64, 72, to 80 mg/L for *F. novicida* or 0.0125, 0.025, 0.05, 0.1, 0.2, 0.5, 1, 2, 3, 4, 8, 16, 32, 40, 48, 56, 64, 72, 80, 96, 104, to 128, mg/L for LVS. Streptomycin resistant derivative strains were obtained by sequentially passaging with the sub-inhibitory concentrations of 0.5, 1, 2, 4, 8, 16, 32, 64, 128, 256, to 512 mg/L for *F. novicida* and LVS. In all cases, the cultures were passaged by alternating between chocolate agar and supplemented BHI broth at 37°C. To confirm resistance stability the isolates were cultured in the absence of antibiotic for 10 passages and alternating agar and broth cultures at 37°C.

### Generation of Growth Curves

*Francisella* strains were cultured for 24 h on chocolate agar and resuspended to an OD_600_ of 0.3 in phosphate buffered saline (PBS). Bacterial suspensions were then diluted 1–10 into either BHI + 1% IsoVitaleX or CDM in a 96-well plate. Growth assays were performed using an Infinite M200 Pro (Tecan Systems, San Jose, CA) microplate reader at 37°C with orbital shaking. All samples were performed in triplicate including medium controls to ensure sterility throughout the experiment as well as to calculate the absorbance of the cultures.

### Minimum Inhibitory Concentration (MIC) Susceptibility Assays

LVS and *F. novicida* strains were pre-cultured for 4–6 h in BHI broth supplemented with 1% IsovitaleX, followed by plating onto chocolate agar and application of the *E*-test strip (bioMérieux, Inc., Durham, NC). *E*-tests were recorded after 24 h for *F. novicida* and 48 h for LVS. For each experiment, three separate *E*-tests were used, and the experiment was repeated three times.

### Genome Sequencing and Analysis

Bacterial genomic DNA was sheared to 400 bp using Covaris LE220 Focused-ultrasonicator (Covaris Inc., Woburn, MA). End repair, A-tailing and ligation of barcoded Illumina adapters were performed on the Apollo 324 automated system, using Prep X Complete ILMN DNA Library Kit (WaferGen Biosystems, Fremont, CA). Libraries were enriched using KAPA Library Amplification Kit (KAPA Biosystems, Wilmington, MA) with 10 cycles of PCR. Libraries were then quantified by TapeStation (Agilent, Santa Clara, CA), normalized and pooled for sequencing. Final pool was quantified by qPCR using KAPA Library Quantification Kit (KAPA Biosystems, Wilmington, MA). Cluster amplification was performed on the cBot with the TruSeq PE Cluster Kit v3-cBot-HS (Illumina Inc., San Diego, CA). Clustered flow cell was sequenced on the HiSeq 2500 instrument, resulting in 2 × 100 bp reads.

Adapters were removed using Cutadapt v1.9dev1 (Martin, 2011), and reads were quality trimmed and filtered with Prinseq v0.20.3 (Schmieder and Edwards, 2011). Reads less than 70 bp in length after trimming and singletons were removed. Reads were aligned to either *Francisella tularensis* subsp *novicida* U112 (NZ_CP009633.1) or *F. tularensis* subsp *holarctica* LVS (NZ_CP009694.1) using Bowtie2 v2.1.0 (Langmead and Salzberg, 2012). For variant calling, we only utilized reads mapped in proper pairs and with mapping quality ≥ 20. Duplicate reads were removed with Picard v1.131 (”Picard Toolkit” 2019; GitHub Repository: Broad Institute) Variants were called using UnifiedGenotyper from GATK v3.5-0-g36282e4 with ploidy set to 1 and genotype likelihoods model set to BOTH (McKenna et al., 2010). Variant effects were annotated using SnpEff v4.2 (Cingolani et al., 2012). Repetitive or low-complexity regions of the genome were identified using custom scripts^1^ and variants called in those regions were filtered. Finally, mutations were confirmed by PCR analysis and Sanger sequencing. Data was deposited into the NCBI database under BioProject ID PRJNA645905.

### Static Biofilm Assay

Bacterial suspensions in PBS were prepared as described above and diluted 1:10 into BHI medium + 1% IsoVitaleX in a 96-well plate. The plates were then incubated statically at 37°C for either 1, 3, 5, or 7 days. Peripheral wells were filled with sterile medium to minimize evaporation over the duration of the experiment. Before staining, the OD_600_ of each well was read to note cell density after which planktonic cells were removed by aspiration and the remaining biofilm was washed 3 times with PBS. When enzymes were used, the biofilm was washed twice with PBS and enzyme (DNase I, proteinase K, or sodium-meta-periodate; Millipore-Sigma, St. Louis, MO) was added at 250 μg/mL in 200 μL of PBS, incubated for 15′ at room temperature, and washed again twice with PBS. In all experiments, the biofilm was fixed for 15′ at room temperature in 100% ethanol. Biofilm was visualized by staining for 15′ with 0.1% crystal violet stain (Sigma-Aldrich, St. Louis, MO) dissolved in H_2_O (w/v) followed 3 PBS washes to remove excess stain. The remaining crystal violet was solubilized in 33% acetic acid (v/v) and the OD_600_ was obtained. In some instances, FM1-43 stain was used to fluorescently label cells within the biofilm. Where indicated, biofilms were stained for 30′ at room temperature using a final concentration of 100 μg/mL FM1-43 after which excess stain was removed by rinsing 3x with water. Fluorescent signal was then recorded using an Infinite M200 Pro (Tecan Systems, San Jose, CA) microplate reader at 472 nm ex/580 nm em set at a gain of 100. When necessary, samples were diluted to ensure OD_600_ or fluorescent readings were within the linear range of the instrument.

### Macrophage Assays

Murine macrophage-like J774A.1 cells (American Type Culture Collection, Manassas, VA) were maintained in Dulbecco’s Modified Eagle’s medium (DMEM) containing 4.5 g/L glucose, L-glutamine and sodium pyruvate supplemented with 10% fetal bovine serum (FBS). Cells were seeded at approximately 2.5 × 10^5^ into 24-well plates and cultured for 24 h at 37°C under 5% CO_2_ to allow a confluent monolayer to form. Prior to the experiment, *F. novicida* was resuspended from 18 h plates to an OD_600_ of ∼0.4 in PBS and diluted 1–5 in pre-warmed tissue culture medium to serve as a bacterial inoculum. Next, tissue culture medium was aspirated from the J774A.1 monolayer, replaced with 200 μL of inoculum (MOI of ∼100:1 confirmed by CFU determination) and incubated for 2 h. After incubation, extracellular bacteria were aspirated, wells were washed 3x with PBS and tissue culture medium containing 25 μg/mL gentamicin was used to replenish each well. At 4 and 24 h, the monolayer of J774A.1 cells was washed 3x with PBS and lysed with 200 μL of sterile water followed by scraping. Cells harvested were immediately suspended in 800 μL of PBS. This suspension was serial diluted and plated on chocolate agar to determine CFU recovery at each time point. For assays with LVS, a 96 well plate format was adapted following the methods described above with minor modifications. J774A.1 cells were seeded at approximately 8 × 10^4^ cells per well in 80 μL. Bacteria were re-suspended for 18 h plates to an OD_600_ of ∼0.4 directly into tissue culture medium, diluted 1:5 into fresh tissue culture medium and 20 μL was added to each well as an inoculum. After 2 h of incubation, 10 mL of fresh tissue culture medium containing gentamicin was added directly to each well without washing at a final concentration of 25 μg/mL. At 4 and 24 h time points, J774A.1 cells were harvested as described above with the exception that 20 μL was used for lysis and immediately re-suspended into 80 μL of PBS. For both *F. novicida* and LVS, the average of three replicate wells was used to determined percent recovery in each experiment. CFU counts were averaged from two plates per well. Results shown were obtained from at least three independent experiments.

### Animal Challenges

Six to eight weeks old female BALB/c mice (obtained from Charles River Laboratories, Frederick, MD) were challenged with LVS or *F. novicida* strains via the intranasal route. Strains were streaked onto chocolate agar and incubated for at 37°C for 2 days (LVS) or 24 h (*F. novicida*), after which the resulting growth was swabbed onto a fresh chocolate agar plate and incubated at 37°C for 24 h. Bacterial growth was resuspended in PBS and cell suspensions diluted to varying doses. Mice were anesthetized with 200 μl of ketamine, acepromazine, and xylazine injected intraperitoneally. These mice were then challenged by intranasal instillation with 50 μL of LVS or *F. novicida* suspended in PBS. Challenge doses were determined by serial dilution in PBS and plating on chocolate agar. Mice were monitored at least twice each day and mortality rates (or euthanasia when moribund) were recorded.

### Ethics Statement

Challenged mice were observed at least twice daily for 21 days for clinical signs of illness. Humane endpoints were used during all studies, and mice were humanely euthanized when moribund according to an endpoint score sheet. Animals were scored on a scale of 0 ± 12: 0 ± 3 = no clinical signs; 4 ± 7 = clinical signs; increase monitoring; 8 ± 12 = distress; euthanize. Those animals receiving a score of 8 ± 12 were humanely euthanized by intraperitoneal injection with 200 μL pentobarbital sodium (Fatal Plus) followed by cervical dislocation. However, even with multiple checks per day, some animals died as a direct result of the infection. Animal research at The United States Army of Medical Research Institute of Infectious Diseases (USAMRIID) was conducted and approved under an Institutional Animal Care and Use Committee (USAMRIID IACUC) in compliance with the Animal Welfare Act, Public Health Service Policy, and other Federal statutes and regulations relating to animals and experiments involving animals. The facility where this research was conducted is accredited by the Association for Assessment and Accreditation of Laboratory Animal Care, International and adheres to principles stated in the Guide for the Care and Use of Laboratory Animals, National Research Council, 2011.

### Statistics

All *in vitro* experiments described in this manuscript were performed independently at least 3 times. Intracellular replication data were analyzed by ANOVA with Dunnett’s *post-hoc* procedure on the ratio of 24–4 h CFU recovery time points, using SAS version 9.4. LD_50_ analysis was determined by the Bayesian probit analysis. Normality was assessed using a Shapiro-Wilk test.

## Results

### Generation of a Surrogate Panel of Antibiotic Resistant *Francisella* Strains

**FIGURE 1:**
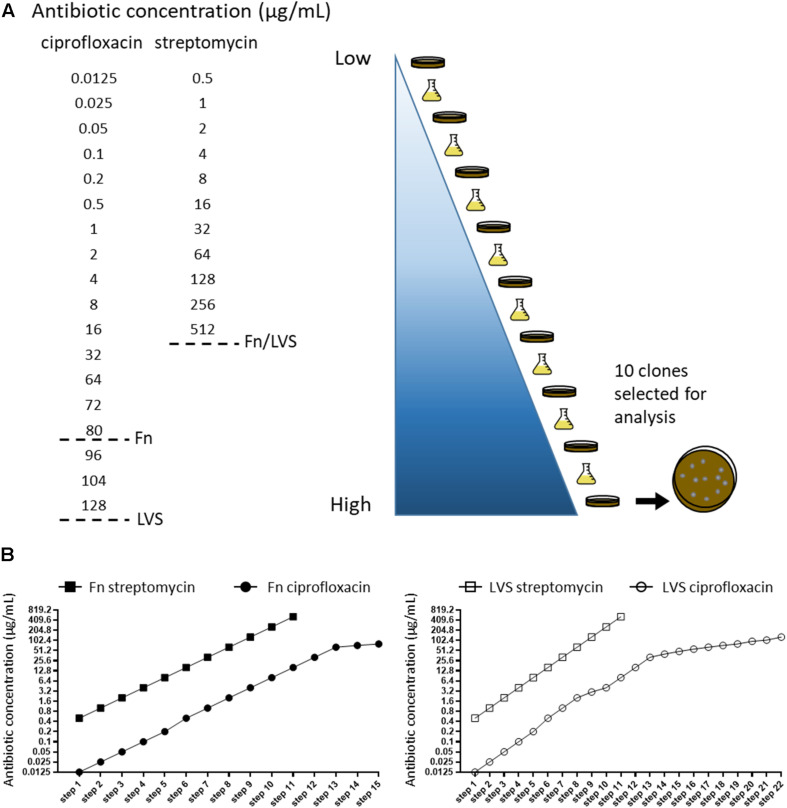
Construction of a surrogate panel of antibiotic resistant *Francisella*. **(A)** Schematic of experimental design. Serial passaging of LVS and Fn was performed on increasing concentrations of ciprofloxacin or streptomycin, alternating between broth and agar. Concentration gradients of each antibiotic are shown from low to high (light blue to dark blue), and dotted lines indicate endpoints for either Fn or LVS passaging. At each endpoint, 10 isolated colonies of either LVS or Fn CipR or StrepR were passaged 10 times without the presence of antibiotic to confirm resistance stability and sequenced. **(B)** Antibiotic concentrations used in each selection step of Fn (left graph) and LVS (right graph) during passaging with ciprofloxacin and streptomycin. Note that LVS required additional intermediate steps of selection on ciprofloxacin, resulting in the 22 selection steps shown.

The acquisition of antibiotic resistance, either natural or engineered, presents a challenge for the development of medical countermeasures. As fluoroquinolones and aminoglycosides often serve as the first line of defense for treatment of tularemia, we sought to characterize a surrogate panel of antibiotic resistant *Francisella* strains in an effort to (1) assess the fitness of such strains; and (2) identify new targets to investigate for the development of vaccines or therapeutics. *F. novicida* U112 and LVS were selected as surrogates due to the relatedness to virulent forms of *F. tularensis* and the ability to work safely under BSL-2 laboratory conditions. To establish a panel of antibiotic resistant strains in these backgrounds, serial passaging in the presence of either ciprofloxacin or streptomycin was conducted in a step-wise manner by increasing the antibiotic concentration in each step (Figure 1A). A previous study compiling data from multiple antibiotic susceptibility studies encompassing > 800 *F. tularensis* strains, reported a range of 0.002–0.125 μg mL^–1^ for ciprofloxacin, with little variation between type A and type B isolates, while a range of 0.064–8 μg mL^–1^ was reported for streptomycin (Caspar and Maurin, 2017). We first tested the MIC values of WT *F. novicida* and LVS strains, then used sub-inhibitory concentrations of 1/4 of the WT MIC for the first selection step. 0.125 μg mL^–1^ ciprofloxacin was used to generate the ciprofloxacin-resistant (CipR) strain panel, and 0.5 μg mL^–1^ streptomycin was used to generate the streptomycin-resistant (StrepR) strain panel. Gradual increase of the antibiotic concentration should select for those genetic adaptations that would enable the cells to replicate in the presence of each respective antibiotic. Both *F. novicida* (Fn) and LVS had increased antibiotic resistance, although the number of passages required to obtain high levels of resistance were dependent on the antibiotic. Passaging of Fn and LVS on streptomycin quickly reached high MIC levels (>1024 μg mL^–1^), after 11 steps. In contrast, cultures in the presence of ciprofloxacin required an additional 3 steps and 6 steps for Fn and LVS, respectively, to achieve an MIC > 32 μg mL^–1^ (Figure 1B). To ensure resistance was stably maintained after selection, 10 CFU of the CipR and StrepR variants were sequentially passaged 10 times in alternating agar and broth cultures without the presence of the respective antibiotic and shown to retain resistance. No differences in growth in BHI/IsovitaleX liquid culture were observed for any of the CipR or StrepR strains tested compared to the parent (data not shown). It is from these antibiotic resistant strains that the surrogate panel was comprised for this study. Strains were designated as follows- Fn or LVS, Cip (ciprofloxacin-resistant) or Strep (streptomycin-resistant), followed by the concentration of antibiotic used in the final step of passaging and the colony number. For example, LVS Cip80-1 indicates LVS passaged on increasing ciprofloxacin to a final concentration of 80 μg mL^–1^, and the first independent colony isolated.

### MIC Determination of CipR and StrepR Resistant LVS and Fn Variants

#### CipR Variants

*E*-tests were carried out to assess final MIC levels of various antibiotics against each LVS or Fn variant. For ciprofloxacin, *E*-tests showed an MIC of 0.008 μg mL^–1^ for the parental Fn strain, and 0.006 μg mL^–1^ for the parental LVS strain. Fn CipR variants exhibited MIC levels of > 32 μg mL^–1^, the maximum concentration on the *E*-tests. In agreement, the LVS Cip128 clones had MIC levels > 32 μg mL^–1^ (Table 1). Additional fluoroquinolones including levofloxacin, ofloxacin, and gatifloxacin were also tested and showed similar patterns of increased resistance among CipR variants as compared to the parental strains (data not shown). CipR variants also exhibited increased resistance to the unrelated antibiotics doxycycline and chloramphenicol in both Fn and LVS (Table 1). Interestingly, all LVS CipR variants became more susceptible to polymyxin B, a membrane-destabilizing antibiotic, while the parent LVS remained resistant to polymyxin B (∼128-384 μg mL^–1^ compared to > 1024 μg mL^–1^ for parent LVS, Table 1). In contrast, the Fn CipR variants displayed slightly increased resistance to polymyxin B, 128 μg mL^–1^, compared to 96 μg mL^–1^ for WT.

#### StrepR Variants

*E*-tests for streptomycin showed MIC values of 2 and 8 μg mL^–1^ for parent LVS and Fn strains, respectively. In contrast, all LVS and Fn StrepR variants showed much higher levels of resistance to streptomycin, MIC > 1,024 μg mL^–1^, at least twofold greater than the concentration of streptomycin used for passaging. Unlike the CipR variants, the acquisition of StrepR did not grant cross protection to related antibiotics, including gentamicin, amikacin, tobramycin, and spectinomycin, as the sensitivities of these variants remained similar to the parental strains (data not shown). Additionally, sensitivity to polymyxin B was unaffected in both Fn and LVS StrepR variants compared to the parental strains (Table 1).

### Genome Sequencing Reveals the Presence of Both On-Pathway and Off-Pathway Mutations in Surrogate Antibiotic Resistant Strains

**FIGURE 2:**
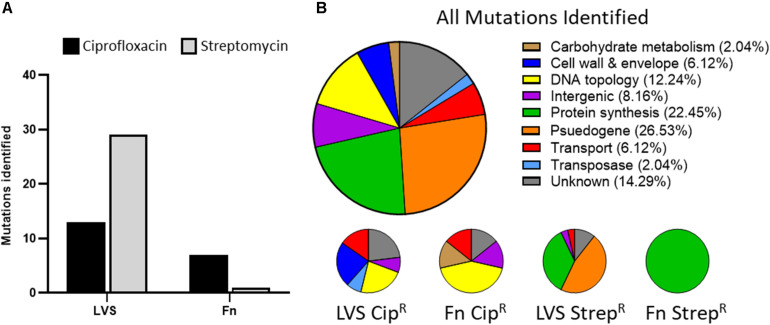
Characterization of mutations harbored within *Francisella* antibiotic resistant surrogates. **(A)** Number of mutations identified in CipR (black bars) and StrepR (gray bars) isolates of LVS and *F. novicida*. **(B)** Annotations of genes found to harbor mutations in all isolates (top pie chart) and LVS or Fn subsets (bottom pie charts) as determined by the prokka genome annotation tool. Percentages of each category are indicated in parentheses.

To identify mutations acquired by *Francisella* during the development of antibiotic resistance, we performed whole genome sequencing on the surrogate strain panel. Sequencing revealed the presence of 49 unique mutations across 25 different genes throughout the surrogate panel, with 20 mutations harbored within CipR strains and 29 harbored within StrepR strains (Supplementary Table S1). Interestingly, the majority of mutations identified were within the LVS background, 41 mutations compared to 8 mutations in the Fn background (Figure 2A). The StrepR LVS isolates harbored about twice as many mutations as the CipR LVS isolates, 28 compared to 13. In contrast, the StrepR Fn isolates harbored only a single mutation, found within the 30S ribosomal protein S12, while CipR Fn isolates contained seven unique mutations (Figure 2A).

To ascribe functional classes to each of these mutations, we next performed Gene Ontology (GO)-term analysis which allowed the mutations and their respective genes to be grouped into nine categories. Figure 2B shows the proportion of mutations occurring in each functional category, with large percentage of mutations found in pseudogenes (∼27%), protein synthesis genes (∼22%), and genes of unknown function (∼14%). Other categories included proteins associated with cell wall and envelope, DNA topology, transport, and intergenic, each accounting for ∼6–12% of the total mutations. Lastly, carbohydrate metabolism and transposases represented ∼2% of the mutations identified. These categories are further broken down into four charts, representing the proportion of mutations found in each panel of strains (LVS CipR, Fn CipR, LVS StrepR, and Fn StrepR as shown in Figure 2B). The DNA topology, cell wall and envelope, and transposase mutations were exclusively contained in the CipR strains, while protein synthesis and pseudogene mutations were exclusively found in the StrepR strains. Notably, these categories can be further divided into on-pathway mutations, present in genes encoding proteins directly targeted by the antibiotic and off-pathway mutations, not known to be directly involved in antibiotic resistance. Supplementary Table S2 lists the patterns of mutations identified across all isolates within the CipR and StrepR panels.

#### On-Pathway Mutations (CipR)

Ciprofloxacin targets DNA replication through the inhibition of DNA gyrase and topoisomerase IV, thereby inhibiting cell division (LeBel, 1988). Consistent with on-pathway mutations, missense mutations were found in genes encoding each of these enzymes in both Fn and LVS CipR strains. All CipR variants of LVS and Fn harbored a single nucleotide polymorphism (SNP) in the *gyrA* allele, resulting in the amino acid substitution of threonine to isoleucine at position 83 of the protein (Table 2). The Fn CipR strains also contained a nearby SNP in *gyrA*, resulting in the amino acid change Asp87Tyr. The *gyrB* allele was only affected in LVS CipR strains, which had a Ser465Tyr mutation. Mutations were also identified in *parC* and *parE* genes, encoding topoisomerase IV subunits A and B, respectively. Unlike *gyrA*, which was mutated in both Fn and LVS, *parC* was only mutated in Fn CipR isolates (Gly81Asp), while *parE* was only mutated in LVS CipR isolates (Ser447Ile). As can be expected, repeated exposure to ciprofloxacin affected genes associated with antibiotic resistance, such as drug efflux pumps. These included an in-frame insertion in a gene encoding for a MexH family multidrug efflux RND transporter, a missense mutation in the gene encoding for an outer membrane efflux protein TolC resulting in Arg453Ser, and a Arg653His substitution in the gene encoding for the AcrB periplasmic accessory protein (Table 2). Interestingly, the *gyrA/B* and *parC/E* mutations were conserved across all colonies of Fn or LVS in which they were identified, while mutations outside of these genes were more scattered across the isolates.

**TABLE 2:** On-pathway mutations identified in CipR and StrepR isolates of *F. novicida* and LVS.

Type	Locus	Protein	Nucleotide change	Amino acid change	Severity
**Fn CipR**					
Missense	AW25_RS02595	GyrA, DNA Gyrase subunit A	248C > T	Thr83Ile	Moderate
Missense	AW25_RS02595	GyrA, DNA Gyrase subunit A	259G > T	Asp87Tyr	Moderate
Missense	AW25_RS07640	ParC, DNA topoisomerase IV subunit A	242G > A	Gly81Asp	Moderate
Insertion (in-frame)	AW25_RS01955	MexH family multidrug efflux RND transporter	785_787dupATC	Asp262_Pro263insHis	Moderate
**LVS CipR**					
Missense	AW21_RS01670	GyrB, DNA Gyrase subunit B	1394C > A	Ser465Tyr	Moderate
Missense	AW21_RS02555	ParE, DNA topoisomerase IV subunit B	1340G > T	Ser447Ile	Moderate
Missense	AW21_RS06550	DNA gyrase subunit A	248C > T	Thr83Ile	Moderate
Frameshift	AW21_RS06550	DNA gyrase subunit A	675delT	Gly225fs	High
Missense	AW21_RS02275	Multidrug transporter AcrB; MMPL family transporter	1958G > A	Arg653His	Moderate
Missense	AW21_RS03270	Outer membrane efflux protein TolC	1357C > A	Arg453Ser	Moderate
**Fn StrepR**					
Missense	AW25_RS08975	30S ribosomal protein S12	128A > G	Lys43Arg	Moderate
**LVS StrepR**					
Missense	AW21_RS04995	30S ribosomal protein S12, RpsL	263A > G	Lys88Arg	Moderate
Missense	AW21_RS04075	Ribosomal RNA small subunit methyltransferase G (RsmG)	10A > T	Met4Leu	Moderate
Frameshift	AW21_RS04075	Ribosomal RNA small subunit methyltransferase G (RsmG)	15delA	Asp6fs	High
Synonymous	AW21_RS04075	Ribosomal RNA small subunit methyltransferase G (RsmG)	60T > C	Thr20Thr	Low
Missense	AW21_RS04075	Ribosomal RNA small subunit methyltransferase G (RsmG)	92T > C	Leu31Pro	Moderate
Frameshift	AW21_RS04075	Ribosomal RNA small subunit methyltransferase G (RsmG)	96_97insA	Leu33fs	High
Missense	AW21_RS04075	Ribosomal RNA small subunit methyltransferase G (RsmG)	236G > T	Gly79Val	Moderate
Nonsense	AW21_RS04075	Ribosomal RNA small subunit methyltransferase G (RsmG)	526G > T	Glu176*	High
Missense	AW21_RS04075	Ribosomal RNA small subunit methyltransferase G (RsmG)	568C > T	Pro190Ser	Moderate
Nonsense	AW21_RS04075	Ribosomal RNA small subunit methyltransferase G (RsmG)	583G > T	Glu195*	High
Missense	AW21_RS00170	MFS transporter	53G > T	Trp18Leu	Moderate

#### Off-Pathway Mutations (CipR)

Mutations deemed off-pathway in CipR Fn strains were both frameshifts which likely resulted in the inactivation of the thiopurine S-methyltransferase (AW25_RS00910) and short-chain dehydrogenase/reductase (SDR) family oxidoreductase (AW25_RS02890) proteins (Table 3). Off-pathway mutations in CipR LVS isolates included a frameshift mutation in *bamB*, encoding a subunit of the beta-barrel assembly machinery, or BAM, complex. Additional mutations were found in genes involved in LPS biosynthesis and transport, *wbtC* (in-frame deletion) and *lptE* (frameshift), and peptidoglycan biosynthesis, *slt* (missense). A frameshift mutation was also identified in the gene encoding for DUF-3573 domain-containing protein FupA (AW21_RS06025). A single mutation was identified in the gene encoding for IS630 family transposase, and the remaining mutations were located in intergenic regions, classified as modifiers in this study, due to the potential for these mutations to modify the expression of downstream genes. In Fn, a cytosine to thymine SNP was identified 35 bp upstream of a IS5 family transposase. In LVS, a cytosine to adenine SNP occurred downstream of a Bcr/CflA family drug resistance efflux transporter, 52 bp upstream of a DUF3573 domain-containing membrane protein. Overall, the mutations found in the Fn CipR panel were present in every isolate, with the exception of a single nucleotide change in an intergenic region in the Cip80-10 strain (Supplementary Table S2). Similarly, the off-pathway mutations in the LVS CipR panel were conserved across all isolates except for those found in *slt*, *lptE*, and an I630 transposase, present in 3–4 isolates out of ten.

**TABLE 3:** Off-pathway mutations identified in CipR and StrepR isolates of *F. novicida* and LVS.

Type	Locus	Protein	Nucleotide change	Amino acid change	Severity
**Fn CipR**					
Frameshift	AW25_RS00910	Thiopurine S-methyltransferase	346_358delCC TAAGATAGCAA	Pro116fs	High
Frameshift	AW25_RS02890	SDR family oxidoreductase	59_60dupCG	Gly21fs	High
Intergenic region	AW25_RS06100-AW25_RS06105	N/A	1278988C > T		Modifier
**Fn StrepR**					
No off-pathway mutations identified					
**LVS CipR**					
Frameshift	AW21_RS02545	Outer membrane protein assembly factor BamB	870delT	Asp290fs	High
Intergenic region	AW21_RS03145-AW21_RS03150	N/A	599763C > A		Modifier
Synonymous	AW21_RS06025	DUF3573 domain-containing protein FupA	105G > C	Gly35Gly	Low
Frameshift	AW21_RS06025	DUF3573 domain-containing protein FupA	107dupC	Leu37fs	High
Missense	AW21_RS06205	Transglycosylase SLT domain protein	1868A > T	Lys623Ile	Moderate
Deletion (in-frame)	AW21_RS06850	NAD-dependent epimerase/dehydratase family protein WbtC	499_510delAAA CTTGCAAAG	Lys167_Lys170del	Moderate
Stop gained	AW21_RS09925	Lipopolysaccharide-assembly family protein LptE	179T > G	Leu60*	High
Frameshift and stop lost	AW21_RS10125	IS630 family transposase, pseudogene	502dupT	Ser168fs	High
**LVS StrepR**					
Intergenic region	AW21_RS00955-AW21_RS10080	10080-IS630 family transposase	164135C > T		Modifier
Frameshift and stop lost	AW21_RS10125	IS630 family transposase, pseudogene	133dupT	Tyr45fs	High
Missense	AW21_RS01780	IS5/IS1182 family transposase, pseudogene	425T > C	Ile142Thr	Moderate
Synonymous	AW21_RS10175	IS630 family transposase, pseudogene	162C > T	Ile54Ile	Low
Frameshift*	AW21_RS06710	Two-component sensor histidine kinase	457 A > T		
	AW21_RS06710	Two-component sensor histidine kinase	458 G > C		
	AW21_RS06710	Two-component sensor histidine kinase	459 T > C		
	AW21_RS06710	Two-component sensor histidine kinase	460 T > C		
	AW21_RS06710	Two-component sensor histidine kinase	462 del GAGGATC		
	AW21_RS06710	Two-component sensor histidine kinase	472 A > G		
	AW21_RS06710	Two-component sensor histidine kinase	473 G > T		
Missense	AW21_RS06710	Two-component sensor histidine kinase	474 del C		
Missense	AW21_RS06960	Recombination factor protein RarA, pseudogene	719C > A	Pro240Gln	Moderate
Nonsense	AW21_RS07120	Aminotransferase, pseudogene	620T > G	Leu207*	High
Missense	AW21_RS08665	Hypothetical protein	725C > T	Ala242Val	Moderate
Missense	AW21_RS08665	Hypothetical protein	737C > G	Thr246Arg	Moderate
Missense	AW21_RS09100	NAD(P)/FAD-dependent oxidoreductase	23G > T	Gly8Val	Moderate

* Mutations identified in AW21_RS06710 ultimately result in frameshift, occurring downstream of the natural frameshift found in the native gene.

#### On-Pathway Mutations (StrepR):

Streptomycin inhibits translation by binding to the 30S ribosomal subunit halting protein synthesis. The 88th residue of this protein was previously found to be important for conferring resistance to streptomycin in *Escherichia coli* (Toivonen et al., 1999). Consistent with this finding, the LVS isolates harbored a missense mutation in *rpsL* (AW21_RS04995) resulting in a Lys88Arg substitution in the 30S ribosomal protein S12 (Table 2). Fn also had a mutated allele of *rspL* (AW25_RS08975) where a Lys43Arg substitution was identified. This mutation was the only one identified in all StrepR Fn strains sequenced. Conversely, the StrepR LVS isolates were some of the most diverse observed during the construction of the antibiotic resistant mutant panels (Table 2). Additional on-pathway mutations arose in the ribosomal RNA small subunit methyltransferase G gene (AW21_RS04075) of LVS StrepR strains. Interestingly, nine unique mutations were identified in this gene with only one being synonymous while the rest were missense mutations (Met4Leu, Leu31Pro, Gly79Val, Pro190Ser) or considered highly detrimental to a functional protein (two frameshifts; Asp6fs, Leu33fs and two stop codon introductions; Glu176^∗^, Glu196^∗^). Lastly, one missense mutation, resulting in Trp18Leu, was found in the gene encoding for the major facilitator superfamily (MFS) transporter (AW21_RS00170), a putative drug efflux protein.

#### Off-Pathway Mutations (StrepR LVS):

The StrepR Fn isolates in our panel only harbored a single mutation that was present in each isolate, the aforementioned missense mutation in *rpsL* (on-pathway), resulting in the substitution of Arg for Lys at position 88. Conversely, the StrepR LVS isolates harbored 28 unique mutations across a variety of loci. Interestingly, a single isolate Fn Strep512-1, harbored a stretch of SNPs and deletions in positions 457–474 of the gene encoding for the two-component sensor histidine kinase (AW21_RS06710). Since this gene is already annotated as a pseudogene, and the mutations occur after the premature stop codon, protein function is likely not impacted. The only two mutations suspected to impact protein function are missense mutations found in the genes encoding for the nicotinamide adenine dinucleotide (phosphate)/ flavin adenine dinucleotide NAD(P)/FAD-dependent oxidoreductase (AW21_RS09100) and hypothetical protein (AW21_RS08665). Unexpectedly, the remaining mutations occurred in pseudogenes or transposases annotated as pseudogenes, as well as a single mutation in an intergenic region (Table 3). These mutations are not likely to affect protein function. Unlike the CipR panel, the vast majority of off-pathway mutations in the StrepR isolates were unique, occurring in only one or a few isolates (Supplementary Table S2).

### The Acquisition of Ciprofloxacin Resistance Alters the Ability of Fn to Form Biofilm *in vitro*

**FIGURE 3:**
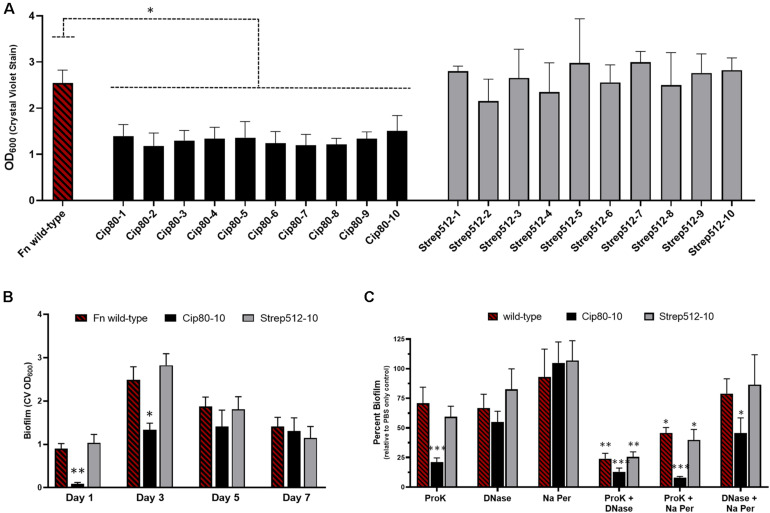
The acquisition of ciprofloxacin resistance delays the formation of biofilm and alters matrix composition. **(A)** Biofilm formation of the *F. novicida* CipR and StrepR surrogate strains as assessed by crystal violet staining after static growth for 3 days at 37°C. **(B)** Time course of biofilm formation for Fn WT compared to representative antibiotic resistant strains Cip80-10 and Strep512-10 as assessed by crystal violet staining after growth for 1, 3, 5, and 7 days. **(C)** Biofilm matrix composition analysis. Biofilms for Fn WT and representative CipR and StrepR strains were grown for 3 days and treated with proteinase K, DNase, sodium metaperiodate, or PBS as a control, then stained with crystal violet to assess biomass. Results are plotted as percent biofilm relative to PBS control. Significance was assessed using a one-way **(A,B)** or two-way ANOVA **(C)**. ^∗^*p* < 0.05, ^∗∗^*p* < 0.01, ^∗∗∗^*p* < 0.001.

The formation of a biofilm signals the transition of a cell from a primarily planktonic lifestyle into a sessile community encased within an extracellular matrix. Life within a biofilm affords a cell protection from environmental stressors and has been attributed to enhanced resistance to oxidative stress and phagocytosis as well as resistance to antibiotics (Shirtliff et al., 2002; Yan and Bassler, 2019). To determine if biofilm formation was altered in the ciprofloxacin and streptomycin resistant strains used in this study, we focused on *F. novicida* antibiotic resistant strains as previous studies have reported that this subspecies readily forms biofilm *in vitro* on a variety of surfaces (Margolis et al., 2010; Zogaj et al., 2012; van Hoek, 2013). To assess biofilm formation, antibiotic resistant strains were grown statically in BHI with IsovitaleX for 3 days at 37°C and subsequently stained with crystal violet. In agreement with growth curve analysis when shaking, static incubation of antibiotic resistant strains yielded a similar optical density to wild-type (data not shown). Interestingly, crystal violet staining revealed that all CipR strains had approximately 50% of the biofilm compared to the parental wild-type strain (Figure 3A). This defect was not observed in StrepR strains, suggesting the phenotype is likely specific to mutations harbored in CipR strains and not an artifact of panel construction. Further, no differences were observed comparing individual strains to each other within the CipR or StrepR panel. Similar results were obtained using FM1-43 as a secondary method of biofilm staining (data not shown).

While Fn CipR strains failed to achieve wild-type levels of biofilm formation, we next wanted to explore the possibility that CipR strains may accrue biofilm at a slower rate giving the appearance of a biofilm defect at 3 days. To address this question, a time course experiment was performed by measuring biofilm formation at 1, 3, 5, and 7 days. Since individual strains within a panel shared a phenotype, Fn Cip80-10 and Fn Strep512-10 were chosen as representative strains for each resistance panel. The time course revealed that peak biofilm in Fn occurred at day 3 and gradually decreased after this time resulting in about 50% of the peak biofilm by day 7 (Figure 3B). Fn Strep512-10 mirrored wild-type and also peaked on day 3 with and decreased to day 7, suggesting that biofilm formation is not affected in these strains. Interestingly, the Fn Cip80-10 strain had a significant decrease in biofilm at both day 1 and 3 compared to wild-type (*p* < 0.01, one-way ANOVA). However, the observed decrease in biofilm did not occur in Fn Cip80-10 after day 3 resulting in similar levels of biofilm by day 7. These results suggest that biofilm development in Fn is a dynamic process as biofilm accrued until day 3, after which the overall amount of biofilm began to decrease, likely due to remodeling processes occurring as the biofilm matures. Further, the acquisition of ciprofloxacin resistance (including either on- or off- target mutations) alters both the ability of Fn biofilm to accrue and remodel as observed in wild-type cells.

We hypothesized that the delayed onset of biofilm development and overall biofilm decrease could be due to an altered matrix composition synthesized by the CipR strains. Given that biofilm is often a mixed polymeric matrix consisting primarily of extracellular DNA, proteins, and polysaccharides, we hypothesized that the ratio of one of these macro-components may be diminished or absent from the matrix. To investigate the composition of the biofilm matrix, Fn biofilm was grown 3 days to achieve peak biofilm then subjected to treatment with the matrix degrading treatments with DNase I, proteinase K, and sodium-meta-periodate, and stained to assess the remaining biofilm. Proteinase K and DNase I had an equal effect on the Fn wild-type biofilm matrix, removing ∼25% of the biomass for each treatment compared to the PBS only treatment while sodium-meta-periodate had little to no effect (Figure 3C). The representative StrepR strain tested displayed no alterations in the biofilm matrix as the treatments yielded results that mirrored wild-type biofilm. Interestingly, proteinase K treatment removed ∼75% of the biofilm formed by the CipR10 mutant (*p* < 0.001, two-way ANOVA), while displaying a similar sensitivity to DNase I as observed in the wild-type biofilm. The susceptibility of the biofilm formed by the CipR mutant to proteinase K treatment suggests that the matrix components in this mutant are altered compared to the wild-type. Indeed, these results are further supported by combinatory treatment of the biofilm matrix with two enzymes simultaneously. In these experiments, the Fn wild-type biofilm was reduced by ∼75% when treated with proteinase K and DNase while the CipR strain was almost completely removed with less than 10% remaining (Figure 3C). These results suggest that polysaccharides do contribute to the Fn biofilm matrix, but likely are a minor component as treatment with both proteinase K and sodium-meta-periodate was able to remove more matrix material than proteinase K alone (25% proteinase K only compared to 50% proteinase K and Na-*m*-periodate). Additionally, combinatory treatment with DNase I and sodium-*m*-periodate shows an increase susceptibility of the CipR biofilm matrix to these treatments compared to wild-type, further suggesting that the matrix is altered in the CipR strains.

### Ciprofloxacin Resistant Isolates Are Impaired for Intracellular Replication in Macrophages

**FIGURE 4:**
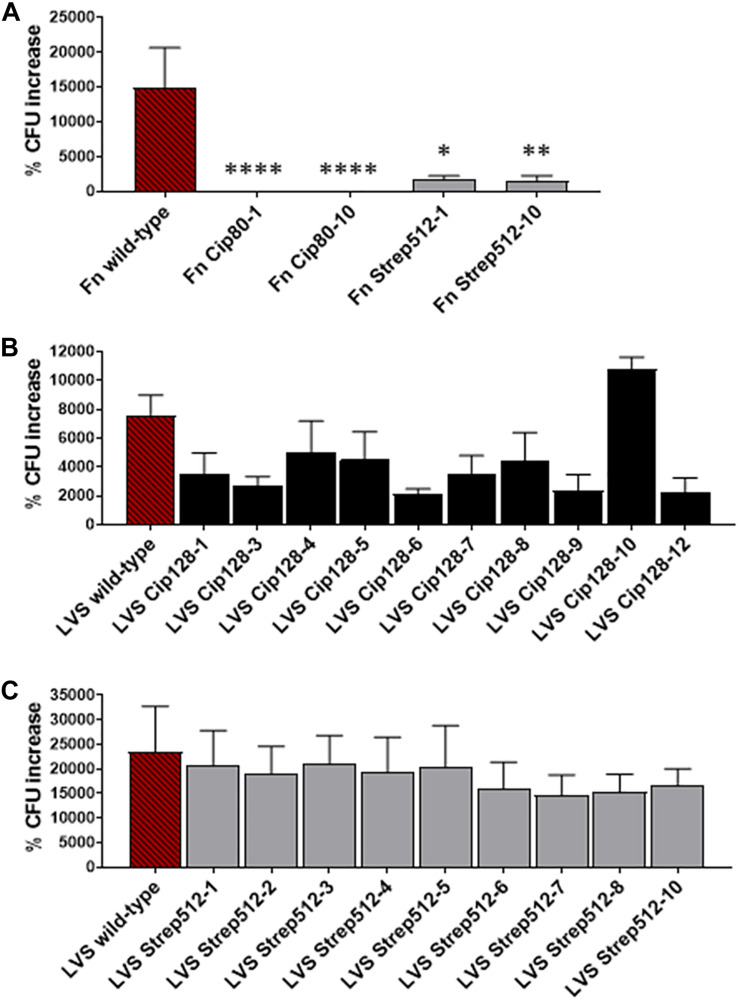
Intracellular replication of CipR and StrepR strains in J774A.1 murine macrophages. Gentamicin protection assays were used to assess intracellular replication of **(A)** *F. novicida* WT and two representative CipR and StrepR isolates, **(B)** the LVS CipR panel, and **(C)** the LVS StrepR panel. Macrophages were infected with *F. novicida* at an MOI ∼100 and cultured in the presence of gentamicin for 4 and 24 h, after which cells were lysed and plated for intracellular bacteria. % CFU recovery of each strain from 24 h compared to 4 h is shown in comparison to the parent *F. novicida* or LVS. Significance was determined using an ANOVA with Dunnett’s *post-hoc* procedure. ^∗^*p* < 0.05, ^∗∗^*p* < 0.01, ^****^p < 0.0001.

The ability of *Francisella* to infect and replicate within macrophages and other host cell types is integral to its pathogenesis in humans and other mammals. In an effort to assess how the development of antibiotic resistance affects fitness in *Francisella*, we performed gentamicin protection assays with the CipR and StrepR surrogate panels using J774A.1 murine macrophage-like cells. Figure 4A shows the percent CFU recovery of *F. novicida* WT and two representative CipR and StrepR isolates, Fn Cip80-1 and Cip80-10, and Fn Strep512-1 and Strep512-10, respectively. Wild-type *F. novicida* exhibited an approximately 2 log increase in the CFU recovered from the 4 to 24 h time-point, with a percent increase of ∼15,000%. In contrast, the Fn CipR strains showed little increase in CFU at 24 h compared to 4 h, and the Fn StrepR strains had a percent CFU increase of ∼1,000%, as shown in Figure 4A. These experiments demonstrate that antibiotic resistant *F. novicida* variants are significantly attenuated for replication in macrophages (*p* < 0.05), a critical host cell reservoir, suggesting these isolates may also be attenuated during infection *in vivo*.

We next tested the entire LVS CipR and StrepR panel for replication in J774A.1 cells using the gentamicin protection assay. In this set of experiments, parent LVS exhibited a % CFU increase of ∼8,000. A general trend with a decrease % CFU recovery was observed with most of the CipR isolates ranging from ∼2,000 to 8,000% (Figure 4B), though not statistically significant. We observed no difference in CFU recovery for all of the LVS StrepR variants (Figure 4C), indicating the mutations acquired during streptomycin resistance acquisition likely have negligible impact on replication within a macrophage for LVS variants.

### Ciprofloxacin and Streptomycin Resistant *Francisella* Are Significantly Attenuated in Mice

To determine how the acquisition of ciprofloxacin and streptomycin resistance affects the virulence of *Francisella* spp., the BALB/c mouse model of intranasal infection was utilized. A panel of Fn and LVS strains was selected for mouse challenges based upon their representative patterns of mutations, as well as their attenuation observed in the above macrophage studies. For Fn variants, which harbored the same mutations across isolates, Fn Cip80-1 and Cip80-10, and Fn Strep512-1 and Strep512-10 were chosen for challenges. For LVS variants, which exhibited more variation in patterns of mutation across isolates, LVS Cip128-3, and Cip128-12 strains were chosen to represent ciprofloxacin passaged strains, while LVS Strep512-1, 4, and 5, were chosen to represent streptomycin-passaged strains.

**FIGURE 5:**
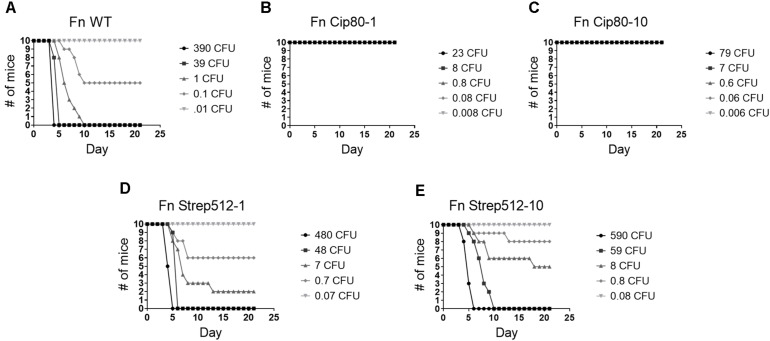
Virulence assessment of *F. novicida* parent **(A)** and select Fn CipR **(B,C)** and StrepR strains **(D,E)** in the BALB/c mouse model of intranasal infection. Mice were inoculated via the intranasal route with 50 μL of varying doses of each strain. Mice were then monitored over the course of 21 days and survival curves plotted. Serial dilutions of bacterial suspensions were plated to enumerate CFUs in challenge doses. LD_50_ values and median time-to-death were calculated and are shown in Table 4.

Both Fn Cip80-1 and Cip80-10 strains were significantly attenuated for virulence, with complete survival of mice at the tested doses of 23 CFU and 79 CFU, respectively, compared to the LD_50_ of ∼0.1 CFU for the parent WT strain (Figures 5A–C and Table 4). In contrast, the Fn Strep512-1 and Strep512-10 strains were slightly (but significantly) attenuated in mice (Figures 5D,E), but no delay in the median time to death was observed compared to the parent Fn strain.

**TABLE 4:** Calculated LD_50_ and median time to death (TTD) for intranasal mouse challenges.

Strain	LD_50_^†^ (95% CL)	*P*-value compared to WT	Median TTD (days)	*P*-value compared to WT
Fn WT	0.1 (.,.)	–	4.0	–
Fn Cip80-1^‡^	>23	<0.0001	>21	<0.0001
Fn Cip80-10^‡^	>79	<0.0001	>21	<0.0001
Fn Strep512-1	1.4 (0.5, 3.6)	0.0004	4.5	N.S.
Fn Strep512-10	4.6 (1.7, 12.3)	<0.0001	5.0	N.S.
LVS WT	172 (52, 701)	–	7.0	–
LVS Cip128-3^‡^	>64,000	<0.0001	>21	<0.0001
LVS Cip128-12^‡^	>253,000	<0.0001	>21	<0.0001
LVS Strep512-1	352 (153, 972)	0.0002	6.0	N.S.
LVS Strep512-4	596 (184, 1615)	<0.0001	6.0	N.S.
LVS Strep512-5	2274 (866, 6313)	<0.0001	6.0	N.S.

*^†^LD_50_ values are estimated under a probit model with 95% confidence limits (CL) by Fieller’s method. P-values were obtained from the Wald Chi-Squared test for each LD_50_ in comparison to the parent strain. Median time to death (TTD) was calculated for the highest dose level tested. ^‡^Comparisons for these strains, in which all groups of mice survived, were made based on the highest dose levels tested.*

**FIGURE 6:**
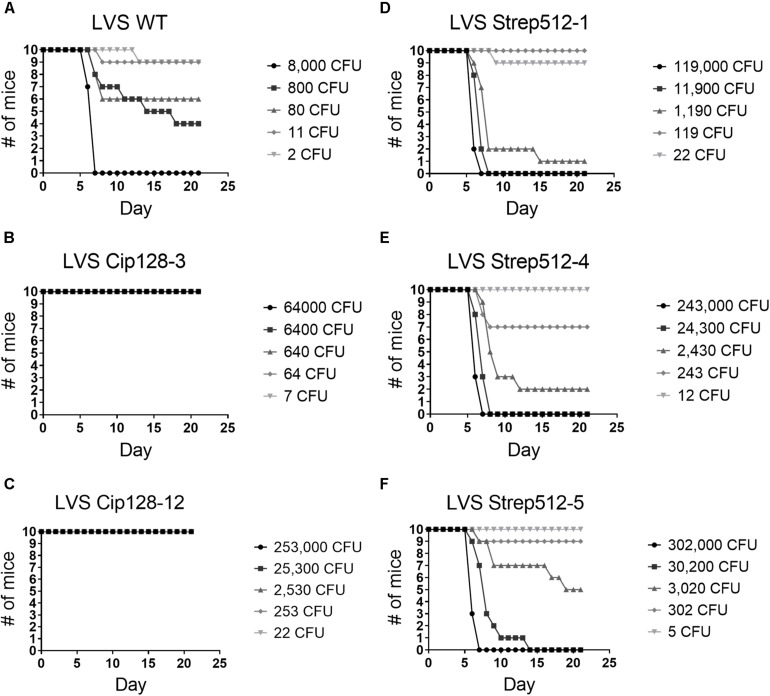
Virulence assessment LVS parent and select CipR and StrepR strains in the BALB/c mouse model of intranasal infection. Mice were infected and survival curves plotted. LD_50_ values and median time-to-death were calculated and are shown in Table 4.

Virulence of CipR and StrepR variants followed a similar pattern in the LVS background. LVS Cip128-3 and Cip128-12 strains were significantly attenuated in the mice, with complete survival even at the highest doses tested, 64,000 CFU and 253,000 CFU, respectively, compared to the calculated LD_50_ of 172 CFU for WT (Figures 6A–C and Table 4). Of the LVS StrepR variants tested, Strep512-1, Strep512-4, and StrepR512-5 had significantly higher LD_50_ values compared to the parent strain; however, the median time to death for all StrepR variants was similar to WT (Figures 6D–F and Table 4). Overall, these results demonstrate that ciprofloxacin and streptomycin resistance leads to decreased virulence in Fn and LVS. Whether this attenuation is a direct result of antibiotic resistance, or due to the auxiliary mutations acquired during development of antibiotic resistance, is an interesting question that remains to be answered.

## Discussion

When cultured in increasing levels of ciprofloxacin or streptomycin, Fn and LVS were able to obtain high levels of resistance after 12 passages. Selecting isolated colonies from these populations allowed for the construction of a ciprofloxacin and streptomycin resistant strain panel featuring a diverse array of mutations across 20 Fn and 20 LVS surrogate strains. Whole genome sequencing revealed that these strains acquired both mutations directly involved in antibiotic resistance (on-pathway) as well as mutations in genes unrelated to antibiotic resistance (off-pathway). Interestingly, when deriving resistance by this natural selection method, many of these surrogate strains were attenuated in a mouse model, most apparent in the CipR strains, suggesting that the evolution of antibiotic resistance to fluoroquinolones and aminoglycosides may come at a cost for *F. tularensis* as virulence was decreased. However, we cannot speak to the fitness of resistant strains derived by other methods or strains passaged without antibiotic selection. With the current data in mind, some of these mutations harbored within these strains are of interest as potential targets for future medical countermeasures. For instance, some of the proteins encoded by the identified virulence factors could be used for future vaccine design as part of a subunit vaccine or in some version of *Francisella* mutation to be used as part of an attenuated live vaccine. Although, a stronger avenue for future biodefense needs, especially, for the enzymatic targets, would be drug design which is discussed in more detail below.

### On-Pathway Mutations Discovered in Ciprofloxacin Resistant Strains

Both *F. novicida* and LVS passaged on ciprofloxacin acquired mutations within the DNA gyrase and topoisomerase IV subunit genes, products of which are known targets of this antibiotic. The subunits of DNA gyrase, *gyrA*, and *gyrB*, and those of the topoisomerase IV, *parC*, and *parE*, share significant sequence homology with a high conservation of amino acids in the quinolone resistance-determining region, or QRDR (Hooper, 2001). Loveless et al. reported several mutations in *gyrA* and *parE* of *F. tularensis* Schu S4 as a result of serial passaging on increasing concentrations of ciprofloxacin (Loveless et al., 2010). Whole genome sequencing of our ciprofloxacin-passaged isolates revealed two mutations in *gyrA* and one mutation in *parC* for Fn CipR strains, as well as one mutation in *gyrA*, one in *gyrB*, and one in *parE* for the LVS CipR strains. All of these mutations led to an amino acid change to the respective protein. Interestingly, the same Thr83Ile mutation was present in *gyrA* of both LVS and *F. novicida* CipR strains; this is a key residue known to be involved in fluoroquinolone resistance across both gram-positive and gram-negative bacteria (Hooper, 2001). This mutation was previously found in a ciprofloxacin-resistant derivative of a *F. tularensis* subsp. *holarctica* clinical isolate, as well as a *F. tularensis* Schu S4, LVS, and *F. novicida* strains passaged on ciprofloxacin (La Scola et al., 2008; Loveless et al., 2010; Sutera et al., 2014). The second mutation found in *gyrA* of the Fn CipR strains was Asp87Tyr, also previously identified as a hotspot for fluoroquinolone resistance in *F. novicida* and LVS, as well as in other species including *Mycobacterium tuberculosis* and *E. coli* (Matrat et al., 2008; Sutera et al., 2014; Jaing et al., 2016; Caspar et al., 2017). Notably, Jaing et al. (2016) observed *gyrA* mutations affecting amino acids 83 and 87 occurring in the same LVS isolate and showed that the presence of both mutations conferred higher levels of ciprofloxacin resistance than each individual mutation (Jaing et al., 2016). Interestingly, the study by Caspar et al. (2017) also found that *F. novicida* developed the first mutation in *gyrB* rather than *gyrA* upon passaging on increasing ciprofloxacin. Our study found the same Ser465Tyr mutation in *gyrB*; however it was only identified in LVS and not Fn CipR variants (Caspar et al., 2017). In most cases, the literature is consistent that gyrase is the primary target of ciprofloxacin, and topoisomerase is the secondary target; typically, a mutation in the gyrase and additional mutation in a topoisomerase gene is required to attain high levels of quinolone resistance. Our study supports this trend, but also adds evidence to suggest that *gyrB* may be the primary target in LVS, while *gyrA* may be the primary target in *F. novicida*. The order of mutations may be dependent on the strain or culture conditions used to derive resistance. Further studies utilizing a range of isolates could shed further light on how these mutations are acquired during antibiotic resistance development.

During passage of Fn on ciprofloxacin concentrations up to 80 μg mL^–1^, we observed high levels of resistance, as shown by *E*-tests (>32 vs. 0.008 for WT). This observation could possibly be explained by the order in which mutations occur in both the gyrase and topoisomerase genes. It has been shown that DNA gyrase is the primary target of quinolone action, and that *parC*-mediated resistance is only obtained in *gyrA* mutants at high fluoroquinolone concentrations (Drlica and Zhao, 1997). All of the Fn CipR variants in our study harbor two mutations in *gyrA* and a single mutation in *parC*. It is likely the *gyrA* mutations were acquired early during passaging, and subsequent passaging on higher concentrations led to the *parC* mutations, resulting in high levels of ciprofloxacin resistance. In general, the presence of two *gyrA* mutations and one *parC* mutation is associated with high levels of quinolone resistance (Vila et al., 1996). Conversely, the LVS CipR strains harbored a single *gyrA* mutation, Thr83Ile also found in Fn CipR, and two additional amino acid changes in *gyrB* and *parE*. In general, mutation of the serine at position 83 in *E. coli* isolates is associated with moderate levels of ciprofloxacin resistance (Drlica and Zhao, 1997). While QRDR mutations typically reduce the fitness of resistant organisms, it has been established that energetically favorable mutations occurring in the QRDR, mainly double-serine mutations, provide favorable fitness to bacteria during resistance development (Fuzi et al., 2017). The Thr83Ile mutation identified in *gyrA* of our study could provide a slight fitness advantage, while all other mutations are expected to cause a loss of fitness (Fuzi et al., 2017).

### Ciprofloxacin Resistant Strains Harbor Multiple Mutations in Efflux Pumps

Several of the mutations found in our resistance panel occurred in genes encoding multidrug efflux pumps. Although not the targets of the antibiotics themselves, these mutations could be expected to alter resistance to either ciprofloxacin or streptomycin. Three mutations occurred in components of resistance-nodulation-cell division (RND) family efflux transporters. The RND efflux transporters are tripartate systems, composed of an outer membrane channel-forming protein, a secondary inner membrane transporter, and a periplasmic adaptor protein that bridges the two membrane proteins. The AcrAB-TolC system of *E. coli* is the most well characterized efflux system, in which TolC forms the outer membrane channel, AcrB functions as the inner membrane transporter, and AcrA is the periplasmic adaptor protein that connects the two (Du et al., 2014).

Previous studies focused on *F. novicida* ciprofloxacin resistance identified mutations in several RND efflux genes, including FTN_1610, encoding an RND efflux transporter, and FTN_1609, encoding the associated periplasmic adaptor subunit (Caspar et al., 2017; Siebert et al., 2019). In our study, we observed a single amino acid change in the outer membrane efflux protein TolC (encoded by AW21_RS03270) of the LVS CipR variants. The mutation resulted in an Arg453Ser substitution located in the outer membrane efflux protein (OEP) domain of *tolC*. The *F. tularensis* genome encodes two orthologs of TolC from *E. coli*, the aforementioned *tolC* gene and *ftlC*, both of which are highly conserved across all three subspecies. Gil et al. (2006) previously demonstrated that *tolC* was necessary for both drug resistance and virulence in a murine model of intradermal infection (Gil et al., 2006).

Additional mutations were identified in the *acrA* and *acrB* homologs upon passaging in ciprofloxacin. All Fn CipR variants contained an insertion of a histidine residue at position 263 of AcrA (AW25_RS01955). A BLAST search of this protein revealed ∼25% similarity to AcrA of *E. coli*, with the mutation present in the domain responsible for interaction with the AcrB subunit. All LVS CipR variants harbored an Arg653His mutation in AcrB (AW21_RS02275). BLAST search showed CmeB from *Campylobacter jejuni* as the closest Protein Data Bank (PDB) entry (97% query cover, ∼29% identity; 5T0O_A). Importantly, a *cmeB* mutant in *C. jejuni* was shown to have enhanced susceptibility to a variety of antibiotics including the fluoroquinolones, and ciprofloxacin specifically was shown to accumulate inside cells of the *cmeB* mutant, further providing support for a role of the *Francisella* AcrB protein in ciprofloxacin resistance (Pumbwe and Piddock, 2002). In contrast, a previous study developed an *acrB* mutant in LVS and showed that, while it was important for resistance to β-lactams and other antibiotics, ciprofloxacin resistance was unaffected (Bina et al., 2008). Moreover, it is unclear whether the AcrA/B subunits described above interact with the TolC or FtlC outer membrane components encoded in the *Francisella* genome, as they can switch adapter subunits.

For both the LVS and Fn CipR variants, mutations found in the efflux pump components could potentially contribute to the slightly higher MIC of doxycycline and chloramphenicol that we observed. These antibiotics would be substrates of these transporters. As fluoroquinolones are substrates of AcrAB-TolC system, mutations in these families of efflux pumps could help the *Francisella* strains to adapt themselves to higher concentrations of ciprofloxacin, resulting in a slight multidrug resistance.

### Off-Pathway Mutations Discovered in Ciprofloxacin Resistant Strains

An interesting mutation that appeared in our study was the presence of a single bp insertion at position 107 in the gene encoding a DUF3573 domain-containing protein, causing a frameshift (AW21_RS06025). This is a unique fusion protein referred to as FupA/B, resulting from a deletion and recombination event between the adjacent genes *fupA* and *fupB*. FupA/B is necessary for iron acquisition in *Francisella*, and interestingly, multiple studies previously found mutations in *fupA/B* in floroquinolone-resistant *Francisella* derivatives (Jaing et al., 2016; Caspar et al., 2017; Chance et al., 2017; Siebert et al., 2019). Jaing et al. (2016) identified multiple *fupA* mutations in ciprofloxacin-resistant LVS isolates, two of which resulted in a premature stop codon and truncated protein product (Jaing et al., 2016). The recent study by Siebert et al. (2019) identified a single nucleotide insertion in the same location as our study, which occurred as a second step event after the development of mutations in the DNA gyrase. They further demonstrated that a *fupA/B* mutant exhibited a threefold increased MIC in comparison to WT, and this was accompanied by increased secretion of outer membrane vesicles and the formation of biofilm (Siebert et al., 2019). Our study showed a decreased biofilm capacity for all of the CipR Fn mutants. However, in agreement with Siebert et al., a screen of the LVS CipR and StrepR panel revealed increased biofilm formation by the CipR strains (Supplementary Figure S1). Perhaps the *fupA* frameshift mutation also identified in our study contributes to the increased outer membrane vesicle secretion and thus increased biofilm capacity.

Two LPS-related genes, *wbtC* and *lptE*, contained mutations resulting in truncated proteins in this study. The *wbtC* gene encodes the NAD-dependent epimerase involved in LPS biosynthesis, while *lptE* encodes an outer membrane lipoprotein that functions in complex with LptD to transport LPS across the outer membrane. Interestingly, the *wbtQ* and *wbtH* genes, also involved in LPS biosynthesis, were found to have mutations resulting in premature stop codons in ciprofloxacin-resistant *F. novicida* (Caspar et al., 2017). Moreover, our laboratory previously reported a mutation in the *kdsD* gene of a ciprofloxacin-resistant derivative of *F. tularensis* Schu S4, which resulted in growth defects, altered LPS profiles, and attenuation in mice (Chance et al., 2017). The high impact mutations reported here and by others support a potential role for the O-antigen in susceptibility to fluoroquinolones.

### On-Pathway Mutations Discovered in Streptomycin Resistant Strains

Streptomycin functions by binding to the 30S subunit of the ribosome, thereby inhibiting protein synthesis (Luzzatto et al., 1968). In our study, both Fn and LVS passaged in streptomycin acquired a mutation in *rpsL*, the gene encoding the 30S ribosomal protein S12. Early publications characterizing high levels of streptomycin resistance in *E. coli* determined that mutation of lysine 42 to asparagine, threonine or arginine in the S12 protein conferred resistance (Funatsu and Wittmann, 1972). This finding was later extended to include lysine 43 in *E. coli* as well as an array of other bacteria after additional work revealed streptomycin interactions directly with the lysine residues at this location of the S12 protein (Sreevatsan et al., 1996; Barnard et al., 2010; Miskinyte and Gordo, 2013). While these amino acid substitutions of RpsL abolish the interaction with streptomycin, the polar substitutions of threonine and asparagine display a restrictive phenotype thereby increasing the fidelity of aminoacyl-tRNA (Sharma et al., 2007). Interestingly, the mutation in *rpsL* resulting in Lys43Arg was one of only two mutations identified in the Fn background. This finding is consistent with the reported non-restrictive phenotype of the Lys42Arg mutation in the S12 protein and may help explain the lack of mutational diversity observed in the Fn StrepR panel. Further, it is possible that the selection pressure applied by streptomycin forced the mutation of *rpsL* early during exposure, and this allele became fixed in the population during subsequent passaging (Heinemann, 1999; Vuilleumier et al., 2008; Baquero et al., 2013). Taken together, these findings potentially explain why relatively few mutations were identified in Fn despite serial passages in streptomycin.

Additional work has identified Lys88 in the S12 protein as a key residue responsible for spontaneous drug resistance in *E. coli*, as frequent mutations K88Q and K88R have been associated with streptomycin resistance (Springer et al., 2001; Benítez-Páez et al., 2014). Consistent with these studies, the K88R mutation in the S12 protein was identified in the LVS StrepR panel in our study. Interestingly, a stretch of single bp changes and deletions were observed in *rsmG*, encoding the ribosomal RNA small subunit methyltransferase G. The *rsmG* allele is annotated as a pseudogene in the LVS genome, and these mutations are located downstream of the premature stop codon, making it highly likely this protein is inactive in our strains. Multiple studies have recently demonstrated that loss of RsmG activity confers low-level streptomycin resistance that gives rise to mutations resulting in high-level resistance (Nishimura et al., 2007; Benítez-Páez et al., 2014). One of these synergistic mutations was identified as the K88R allele of the S12 protein (Benítez-Páez et al., 2014).

Several LVS StrepR variants were found to contain a Trp18Leu substitution in the MFS transporter. MFS transporters facilitate the transfer of a wide range of small molecules substrates across the cell membrane including amino acids, sugars, oxyanions, and large peptides. However, in bacteria, MFS transporters are mostly credited as nutrient transporters and drug efflux pumps (Schuldiner, 2018; Kumar et al., 2020). Previous work has shown that MFS transporters play an essential role in *F. tularensis* virulence by influencing nutrient acquisition within a host cell, and that inactivation of key MFS transporters results in attention *in vivo* in both Type A and B strains (Pérez et al., 2016; Balzano et al., 2018). Given the mild attenuation observed in the StrepR LVS strains, it is likely that this transporter functions as an efflux pump, though further work is required to identify the substrate of the MFS transporter identified in these strains.

### Off-Pathway Mutations Discovered in Streptomycin Resistant Strains

No off-pathway mutations were found in the Fn StrepR surrogate panel. However, the LVS StrepR panel contained a multitude of off-pathway mutations, the majority of which were found in pseudogenes and transposases (discussed below). Two exceptions were mutations in the NAD(P)/FAD-dependent oxidoreductase and a hypothetical protein, neither of which have been previously reported to be involved in antibiotic resistance. Several LVS StrepR mutations were found in or nearby transposase genes, including four in IS630 family transposases and one in an IS5/IS1182 family transposase, all of which were annotated as pseudogenes. Moreover, the LVS CipR panel contained a frameshift in the gene encoding for an IS630 family transposase, similar to several LVS StrepR strains. The complete genome sequence of *F. tularensis* Schu S4 was reported to have an abundance of IS elements, the most common of which were IS630 family (ISFtu1) with 50 copies, and IS5 family (ISFtu2) with 16 copies (Larsson et al., 2005). It is possible that mutations frequently occurred in the IS elements because they are prevalent in the genome or that these regions tend to have higher mutation frequencies in general.

### Ciprofloxacin Resistance Decreases the Biofilm Forming Capacity of Fn

The prevailing model is that biofilm formation allows *Francisella* to adapt and persist in an aquatic environment or vector reservoir (Mahajan et al., 2011; van Hoek, 2013). It is well established that biofilm enhances the ability of bacteria to cope with antibiotic stress (Chung et al., 2014; Siebert et al., 2019, 2020). Opposing this dogma, a link between quinolone resistance and biofilm forming capacity has been identified in several gram-negative bacteria including *E. coli, Klebsiella pneumonia*, *Pseudomonas aeruginosa*, and *Salmonella enterica*, with most studies reporting a decreased biofilm capacity as resistance to quinolones increases (Fàbrega et al., 2014; Cepas et al., 2018). In *Salmonella*, this has been attributed to decreased expression of type 1 fimbriae and the *csg* loci encoding curli, though *Francisella* appears to lack both of these systems (Vila and Soto, 2012; Fàbrega et al., 2014). Despite this, our results are consistent with these studies as we found that Fn CipR mutants displayed a delay in biofilm development and failed to reach wild-type levels. Ahmed and colleagues found that passaging *P. aeruginosa* in low-levels of ciprofloxacin either planktonically or in a biofilm resulted in CipR strains harboring different mutations, likely due to differences in selection pressures applied during a given lifestyle (Ahmed et al., 2018). Mutations identified in our study that overlap with Ahmed et al. (2018) were found in the Mex transporter family, *gyrA* and *gyrB*. Interestingly, the latter of these mutations were only identified in planktonically grown cultures, which is similar to how the strain panel in this study was constructed. Additional work in *P. aeruginosa* has suggested that these classic fluoroquinolone mutations in *gyrA* are permitted to occur due to secondary mutations in other genes, which in at least one instance has come at the cost of enzymes involved in cyclic-di-GMP signaling altering biofilm formation (Wong et al., 2012). Indeed, it has been convincingly demonstrated that cyclic-di-GMP is necessary for stimulating biofilm formation in *F. novicida* (Zogaj et al., 2012). In contrast, LVS lacks this signaling pathway, which likely accounts for its decreased biofilm capacity relative to *F. novicida* (Zogaj et al., 2012; van Hoek, 2013). With this in mind, further work is needed to investigate this possibility as our data show that Fn CipR strains can still form biofilm, but in a reduced capacity under an altered membrane composition.

### Antibiotic Resistance Obtained by Natural Selection Leads to Attenuated Virulence in Fn and LVS

In our study, we sought to determine the effect of antibiotic resistance acquisition by serial passaging on virulence of the LVS and Fn strains using a macrophage replication assay and a murine model of intranasal infection. We showed that both CipR and StrepR *F. novicida* were significantly altered in replication in macrophages. However, CipR LVS strains had more variable levels of replication but most displayed a general trend for decreased intracellular replication. Interestingly, streptomycin resistance in LVS had no effect on replication in macrophages. Virulence studies using BALB/c mice showed similar patterns of attenuation as in the macrophage model, with Fn CipR isolates, and to a lesser extent Fn StrepR strains, showing significant attenuation. Similarly, the LVS CipR strains exhibited substantially more attenuation in mice, while the StrepR strains were mildly affected. Overall, these studies demonstrate that CipR strains were much more attenuated in macrophages and mice than StrepR strains, indicating the development of ciprofloxacin resistance obtained as described here may have broader impacts on virulence than that of streptomycin. A recent study demonstrated that deletion of TolC had significant impacts on both resistance profiles and virulence of *F. tularensis* SchuS4, suggesting a link between both antibiotic resistance and pathogenicity (Kopping et al., 2019). Similarly, the AcrB RND efflux protein from LVS was shown to be required for both β-lactam resistance and full virulence in mice (Bina et al., 2008). These studies support a dual role for multi-drug resistant efflux pumps in antibiotic resistance and virulence. The mutations in AcrA/B and TolC found in our study could account for some of the attenuation seen in the Fn and LVS CipR strains, although the strains in our panel contained single amino acid substitutions that increased resistance, as opposed to genetic deletions that increased antibiotic susceptibility of the strains. To our knowledge, this is one of the first studies to examine the link between naturally acquired antibiotic resistance and virulence in *Francisella* spp.

The acquisition of antibiotic resistance in bacteria is often accompanied by defects in fitness (Beceiro et al., 2013) which may explain the attenuation observed in our CipR and StrepR panel. However, this occurrence is highly species-dependent, with fluoroquinolone resistance coming at a cost to some organisms (i.e., *Salmonella enterica* and *Acinetobacter baumannii*), but not others (i.e., *Pseudomonas aeruginosa* and *E. coli*). The fitness cost also depends on the type of mutation. For example, it was shown in *E. coli* that streptomycin resistance acquired through mutations in ribosomal methyltransferases has no fitness cost, while ribosomal mutations come at a cost (Andersson and Levin, 1999; Gutierrez et al., 2012). In an elegant study, Marcusson et al. (2009) constructed 28 isogenic *E. coli* strains with combinations of up to five fluoroquinolone resistance mutations to examine the relationship between resistance and fitness. They found that fitness was affected by the nature of the mutations involved, rather than the number of mutations, and that it was possible for strains to gain high resistance levels with little compromise in fitness (Marcusson et al., 2009). Interestingly, a review by Fuzi et al. (2020), highlights that recent clonal expansion of diverse multi-drug resistant pathogens, including hospital-associated methicillin-resistant *Staphylococcus aureus* and extended spectrum β-lactamase-producing *Klebsiella pneumoniae* and *E. coli*, was likely driven by increased use of fluoroquinolones in recent decades (Fuzi et al., 2020). The studies summarized within demonstrate that fluoroquinolone resistance typically result in QRDR mutations or enhanced efflux activity that come at a fitness cost. However, major clones that had evolved energetically favorable QRDR mutations, were able to regain fitness and replace the minor clones (Fuzi et al., 2017, 2020).

It is currently unknown how these resistance mechanisms specifically affect fitness and virulence in *Francisella* spp. Alternatively, the different off-pathway mutations identified in CipR and StrepR strains could account for this. Indeed, several mutations were found in known or putative virulence factors, including Slt (cell wall biosynthesis), WbtC and LptE (LPS biosynthesis), and TolC (drug transport). It is important to note that the attenuation observed in some resistant isolates may be the result of passage in culture giving rise to attenuating mutations, rather than a result of the antibiotic selection itself. However, no overlapping mutations were found in CipR and StrepR strains, which suggests this is likely not the case. A recent investigation into the amplification of low frequency mutations during the passaging of *F. tularensis* without antibiotics further argues against this concept, since SNPs and small deletions were mainly found in capsule synthesis genes (Dwibedi et al., 2020). Further studies utilizing single mutants are required to determine the extent that the individual mutations contribute to attenuation.

### Antibiotic Resistant Mutations for Identification of Future Drug Targets

A major strength of this study and other studies focused on deriving antibiotic resistant isolates is that off-target mutations are often important in cellular functions and can be sensitive to environmental stressors, like antibiotic selection, allowing for the identification of new drug targets. As a proof of principle, the *slt* gene identified in this study was found to be necessary for maintaining cell growth, morphology, and virulence and morphology of *F. novicida* (Bachert et al., 2019). Interestingly, the *slt* gene could be mutated in *F. novicida*, but not in LVS or *F. tularensis* Schu S4 strains, supporting *slt* as an essential gene in these species and an ideal therapeutic target (Bachert et al., 2019; Ireland et al., 2019). The mutation identified here likely does not inactivate the enzyme, given that it is essential in LVS, and that the mutation was found outside of the cluster of residues thought to be required for sugar binding and catalytic activity. Previous work in our laboratory also identified the presence of a frameshift mutation in the *kdsD* gene in a ciprofloxacin resistant variant of *F. tularensi*s Schu S4. The *kdsD* gene encodes an enzyme involved in LPS biosynthesis, and a Schu S4 mutant was attenuated in mice (Chance et al., 2017). Current studies in our laboratory are aimed at assessing the therapeutic potential of KdsD. Numerous other studies have identified potential therapeutic targets for tularemia via the development of antibiotic resistant panels, including the FupA/B fusion protein (Siebert et al., 2019), WbtQ and WbtH proteins involved in LPS biosynthesis, and FTN_1029, encoding a protein involved in isoprenoid biosynthesis, necessary for cell wall synthesis and membrane fluidity (Caspar et al., 2017). These experiments enable us to understand how *Francisella* species and perhaps other gram-negative pathogens may respond to antibiotics and allow us to develop new strategies to overcome antibiotic resistance.

## Data Availability Statement

The datasets presented in this study can be found in online repositories. The names of the repository/repositories and accession number(s) can be found below: https://www.ncbi.nlm.nih.gov/genbank/, SAMN15518680 SAMN15-518681 SAMN15518682 SAMN15518683 SAMN15518684 SA-MN15518685 SAMN15518686 SAMN15518687 SAMN15518688 SAMN15518689 SAMN15518690 SAMN15518691 SAMN155-18692 SAMN15518693 SAMN15518694 SAMN15518695 SA-MN15518696 SAMN15518697 SAMN15518698 SAMN155186-99 SAMN15518700 LSAMN15518701 SAMN15518702 SA-MN15518703 SAMN15518704 SAMN15518705 SAMN15518706 SAMN15518707 SAMN15518708 SAMN15518709.

## Ethics Statement

Animal research at the United States Army Medical Research Institute of Infectious Diseases (USAMRIID) was approved and conducted under an Institutional Animal Care and Use Committee (USAMRIID IACUC) in compliance with the Animal Welfare Act, Public Health Service Policy, and other Federal statutes and regulations relating to animals and experiments involving animals. The facility where this research was conducted is accredited by the Association for Assessment and Accreditation of Laboratory Animal Care, International and adheres to principles stated in the Guide for the Care and Use of Laboratory Animals, National Research Council, 2011.

## Author Contributions

FB, BB, KM, and JB contributed conception and design of the study and wrote the manuscript. FB, BB, KM, RT, GK, SL, CK, CC, JL, and JB participated in the experimentation and acquisition of data. FB, BB, KM, SL, JL, and JB were involved in the analysis or interpretation of data for the work. All authors contributed to manuscript revision, read and approved the submitted version.

## Disclaimer

Opinions, interpretations, conclusions, and recommendations are those of the authors and are not necessarily endorsed by the U.S. Army.

## Conflict of Interest

The authors declare that the research was conducted in the absence of any commercial or financial relationships that could be construed as a potential conflict of interest.

## Footnotes

1 https://github.com/lovettse/Seq_utils/blob/master/genotyping/

## References

[B1] Ahmed M. N. Porse A. Sommer M. O. A. Høiby N. Ciofu O. (2018). Evolution of antibiotic resistance in biofilm and planktonic *Pseudomonas aeruginosa* populations exposed to subinhibitory levels of ciprofloxacin. *Antimicrob. Agents Chemother.* 62: e00320-18. 10.1128/AAC.00320-18 PMC6105853 29760140

[B2] Andersson D. I. Levin B. R. (1999). The biological cost of antibiotic resistance. *Curr. Opin. Microbiol.* 2 489–493. 10.1016/s1369-5274(99)00005-3 10508723

[B3] Bachert B. A. Biryukov S. S. Chua J. Rodriguez S. A. Toothman R. G. Jr. (2019). A *Francisella novicida* mutant, lacking the soluble lytic transglycosylase Slt, exhibits defects in both growth and virulence. *Front. Microbiol.* 10:1343. 10.3389/fmicb.2019.01343 31258523 PMC6587636

[B4] Balzano P. M. Cunningham A. L. Grassel C. Barry E. M. (2018). Deletion of the major facilitator superfamily transporter *fptB* alters host cell interactions and attenuates virulence of type A *Francisella tularensis*. *Infect. Immun.* 86:e00832-17. 10.1128/IAI.00832-17 PMC5820938 29311235

[B5] Baquero F. Tedim A. P. Coque T. M. (2013). Antibiotic resistance shaping multi-level population biology of bacteria. *Front. Microbiol.* 4:15. 10.3389/fmicb.2013.00015 23508522 PMC3589745

[B6] Barnard A. M. L. Simpson N. J. L. Lilley K. S. Salmond G. P. C. (2010). Mutations in *rpsL* that confer streptomycin resistance show pleiotropic effects on virulence and the production of a carbapenem antibiotic in *Erwinia carotovora*. *Microbiology* 156 1030–1039. 10.1099/mic.0.034595-0 20056700

[B7] Barras V. Greub G. (2014). History of biological warfare and bioterrorism. *Clin. Microbiol. Infect.* 20 497–502. 10.1111/1469-0691.12706 24894605

[B8] Beceiro A. Tomás M. Bou G. (2013). Antimicrobial resistance and virulence: a successful or deleterious association in the bacterial world? *Clin. Microbiol. Rev.* 26 185–230. 10.1128/cmr.00059-12 23554414 PMC3623377

[B9] Benítez-Páez A. Cárdenas-Brito S. Corredor M. Villarroya M. Armengod M. E. (2014). Impairing methylations at ribosome RNA, a point mutation-dependent strategy for aminoglycoside resistance: the *rsmG* case. *Biomedica* 34(Suppl. 1), 41–49. 24968035 10.1590/S0120-41572014000500006

[B10] Bina X. R. Lavine C. L. Miller M. A. Bina J. E. (2008). The AcrAB RND efflux system from the live vaccine strain of *Francisella tularensis* is a multiple drug efflux system that is required for virulence in mice. *FEMS Microbiol. Lett.* 279 226–233. 10.1111/j.1574-6968.2007.01033.x 18179581

[B11] Caspar Y. Hennebique A. Maurin M. (2018). Antibiotic susceptibility of *Francisella tularensis* subsp. holarctica strains isolated from tularaemia patients in France between 2006 and 2016. *J. Antimicrob. Chemother.* 73 687–691. 10.1093/jac/dkx460 29253157

[B12] Caspar Y. Maurin M. (2017). *Francisella tularensis* susceptibility to antibiotics: a comprehensive review of the data obtained *in vitro* and in animal models. *Front. Cell Infect. Microbiol.* 7:122. 10.3389/fcimb.2017.00122 28443249 PMC5386985

[B13] Caspar Y. Siebert C. Sutera V. Villers C. Aubry A. Mayer C. (2017). Functional characterization of the DNA gyrases in fluoroquinolone-resistant mutants of *Francisella novicida*. *Antimicrob. Agents Chemother.* 61:e02277-16. 10.1128/AAC.02277-16 28167561 PMC5365679

[B14] Cepas V. López Y. Muñoz E. Rolo D. Ardanuy C. Martí S. (2018). Relationship between biofilm formation and antimicrobial resistance in Gram-negative bacteria. *Microb. Drug Resist.* 25 72–79. 10.1089/mdr.2018.0027 30142035

[B15] Chamberlain R. E. (1965). Evaluation of live tularemia vaccine prepared in a chemically defined medium. *Appl. Microbiol.* 13 232–235. 10.1128/aem.13.2.232-235.1965 14325885 PMC1058227

[B16] Champion A. E. Catanzaro K. C. F. Bandara A. B. Inzana T. J. (2019). Formation of the *Francisella tularensis* biofilm is affected by cell surface glycosylation, growth medium, and a glucan exopolysaccharide. *Sci. Rep.* 9:12252. 10.1038/s41598-019-48697-x PMC6706388 31439876

[B17] Chance T. Chua J. Toothman R. G. Ladner J. T. Nuss J. E. Raymond J. L. (2017). A spontaneous mutation in *kdsD*, a biosynthesis gene for 3 Deoxy-D-manno-Octulosonic Acid, occurred in a ciprofloxacin resistant strain of *Francisella tularensis* and caused a high level of attenuation in murine models of tularemia. *PLoS One* 12:e0174106. 10.1371/journal.pone.0174106 28328947 PMC5362203

[B18] Chung M. C. Dean S. Marakasova E. S. Nwabueze A. O. Van Hoek M. L. (2014). Chitinases are negative regulators of *Francisella novicida* biofilms. *PLoS One* 9:e93119. 10.1371/journal.pone.0093119 24664176 PMC3963990

[B19] Cingolani P. Platts A. Wang, Le L. Coon M. Nguyen T. (2012). A program for annotating and predicting the effects of single nucleotide polymorphisms, SnpEff: SNPs in the genome of *Drosophila melanogaster* strain w1118; iso-2; iso-3. *Fly* 6 80–92. 10.4161/fly.19695 22728672 PMC3679285

[B20] Costerton J. W. Stewart P. S. Greenberg E. P. (1999). Bacterial biofilms: a common cause of persistent infections. *Science* 284 1318–1322. 10.1126/science.284.5418.1318 10334980

[B21] Dennis D. T. Inglesby T. V. Henderson D. A. Bartlett J. G. Ascher M. S. Eitzen E. (2001). Tularemia as a biological weapon: medical and public health management. *JAMA* 285 2763–2773. 10.1001/jama.285.21.2763 11386933

[B22] Drlica K. Zhao X. (1997). DNA gyrase, topoisomerase IV, and the 4-quinolones. *Microbiol. Mol. Biol. Rev.* 61 377–392. 10.1128/.61.3.377-392.1997 9293187 PMC232616

[B23] Du D. Wang Z. James N. R. Voss J. E. Klimont E. Ohene-Agyei T. (2014). Structure of the AcrAB-TolC multidrug efflux pump. *Nature* 509 512–515. 24747401 10.1038/nature13205 PMC4361902

[B24] Dwibedi C. Larsson P. Ahlinder J. Lindgren P. Myrtennäs K. Granberg M. (2020). Biological amplification of low frequency mutations unravels laboratory culture history of the bio-threat agent *Francisella tularensis*. *Forensic Sci. Int. Genet.* 45:102230. 10.1016/j.fsigen.2019.102230 31924594

[B25] Eigelsbach H. T. Downs C. M. (1961). Prophylactic effectiveness of live and killed tularemia vaccines. I. Production of vaccine and evaluation in the white mouse and guinea pig. *J. Immunol.* 87 415–425. 13889609

[B26] Ellis J. Oyston P. C. Green M. Titball R. W. (2002). Tularemia. *Clin. Microbiol. Rev.* 15 631–646. 12364373 10.1128/CMR.15.4.631-646.2002 PMC126859

[B27] Fàbrega A. Soto S. M. Ballesté-Delpierre C. Fernández-Orth D. Jiménez, De Anta M. T. (2014). Impact of quinolone-resistance acquisition on biofilm production and fitness in *Salmonella enterica*. *J. Antimicrob. Chemother.* 69 1815–1824. 10.1093/jac/dku078 24706735

[B28] Funatsu G. Wittmann H. G. (1972). Ribosomal proteins: XXXIII. Location of amino-acid replacements in protein S12 isolated from *Escherichia coli* mutants resistant to streptomycin. *J. Mol. Biol.* 68 547–550. 4560854 10.1016/0022-2836(72)90108-8

[B29] Fuzi M. Rodriguez Baño J. Toth A. (2020). Global evolution of pathogenic bacteria with extensive use of fluoroquinolone agents. *Front. Microbiol.* 11:271. 10.3389/fmicb.2020.00271 32158437 PMC7052298

[B30] Fuzi M. Szabo D. Csercsik R. (2017). Double-serine fluoroquinolone resistance mutations advance major international clones and lineages of various multi-drug resistant bacteria. *Front. Microbiol.* 8:2261. 10.3389/fmicb.2017.02261 29250038 PMC5715326

[B31] Gil H. Platz G. J. Forestal C. A. Monfett M. Bakshi C. S. Sellati T. J. (2006). Deletion of TolC orthologs in *Francisella tularensis* identifies roles in multidrug resistance and virulence. *Proc. Natl. Acad. Sci. U.S.A.* 103 12897–12902. 10.1073/pnas.0602582103 16908853 PMC1568944

[B32] Gutierrez B. Escudero J. A. San Millan A. Hidalgo L. Carrilero L. Ovejero C. M. (2012). Fitness cost and interference of Arm/Rmt aminoglycoside resistance with the RsmF housekeeping methyltransferases. *Antimicrob. Agents Chemother.* 56 2335–2341. 10.1128/aac.06066-11 22330907 PMC3346654

[B33] Hall C. W. Mah T. F. (2017). Molecular mechanisms of biofilm-based antibiotic resistance and tolerance in pathogenic bacteria. *FEMS Microbiol. Rev.* 41 276–301. 10.1093/femsre/fux010 28369412

[B34] Heine H. S. Miller L. Halasohoris S. Purcell B. K. (2017). *In vitro* antibiotic susceptibilities of *Francisella tularensis* determined by broth microdilution following CLSI methods. *Antimicrob. Agents Chemother.* 61 e612–e617. 10.1128/AAC.00612-17 PMC5571322 28674048

[B35] Heinemann J. A. (1999). How antibiotics cause antibiotic resistance. *Drug Discov. Today* 4 72–79. 10.1016/s1359-6446(98)01294-x 10234159

[B36] Hepburn M. J. Simpson A. J. (2008). Tularemia: current diagnosis and treatment options. *Expert Rev. Anti Infect. Ther.* 6 231–240. 10.1586/14787210.6.2.231 18380605

[B37] Hooper D. C. (2001). Mechanisms of action of antimicrobials: focus on fluoroquinolones. *Clin. Infect. Dis.* 32(Suppl. 1), S9–S15. 11249823 10.1086/319370

[B38] Hopla C. E. (1974). The ecology of tularemia. *Adv. Vet. Sci. Comp. Med.* 18 25–53. 4419176

[B39] Hornick R. B. Eigelsbach H. T. (1966). Aerogenic immunization of man with live Tularemia vaccine. *Bacteriol. Rev.* 30 532–538. 10.1128/mmbr.30.3.532-538.1966 5917334 PMC378235

[B40] Ireland P. M. Bullifent H. L. Senior N. J. Southern S. J. Yang Z. R. Ireland R. E. (2019). Global analysis of genes essential for *Francisella tularensis* Schu S4 growth *in vitro* and for fitness during competitive infection of Fischer 344 rats. *J. Bacteriol.* 201:e00630-18. 10.1128/JB.00630-18 PMC6416918 30642993

[B41] Jacoby G. A. (2005). Mechanisms of resistance to quinolones. *Clin. Infect. Dis.* 41 S120–S126. 15942878 10.1086/428052

[B42] Jaing C. J. Mcloughlin K. S. Thissen J. B. Zemla A. Gardner S. N. Vergez L. M. (2016). Identification of genome-wide mutations in ciprofloxacin-resistant *F. tularensis* LVS using whole genome tiling arrays and next generation sequencing. *PLoS One* 11:e0163458. 10.1371/journal.pone.0163458 27668749 PMC5036845

[B43] Jellison W. L. Parker R. R. (1945). Rodents, rabbits and tularemia in North America: some zoological and epidemiological considerations. *Am. J. Trop.* s1-25 349–362. 10.4269/ajtmh.1945.s1-25.349

[B44] Johansson A. Berglund L. Sjostedt A. Tarnvik A. (2001). Ciprofloxacin for treatment of tularemia. *Clin. Infect. Dis.* 33 267–268. 10.1086/321825 11418893

[B45] Jones R. M. Nicas M. Hubbard A. Sylvester M. D. Reingold A. (2005). The infectious dose of *Francisella tularensis* (tularemia). *Appl. Biosaf.* 10 227–239.

[B46] Kingry L. C. Petersen J. M. (2014). Comparative review of *Francisella tularensis* and *Francisella novicida*. *Front. Cell Infect. Microbiol.* 4:35. 10.3389/fcimb.2014.00035 24660164 PMC3952080

[B47] Kopping E. J. Doyle C. R. Sampath V. Thanassi D. G. (2019). Contributions of TolC orthologs to *Francisella tularensis* Schu S4 multidrug resistance, modulation of host cell responses, and virulence. *Infect. Immun.* 87:e00823-18. 10.1128/IAI.00823-18 30670554 PMC6434128

[B48] Kumar S. Lekshmi M. Parvathi A. Ojha M. Wenzel N. Varela M. F. (2020). Functional and structural roles of the major facilitator superfamily bacterial multidrug efflux pumps. *Microorganisms* 8:266. 10.3390/microorganisms8020266 32079127 PMC7074785

[B49] La Scola B. Elkarkouri K. Li W. Wahab T. Fournous G. Rolain J. M. (2008). Rapid comparative genomic analysis for clinical microbiology: the *Francisella tularensis* paradigm. *Genome Res.* 18 742–750. 10.1101/gr.071266.107 18407970 PMC2336804

[B50] Langmead B. Salzberg S. L. (2012). Fast gapped-read alignment with Bowtie 2. *Nat. Methods* 9 357–359. 10.1038/nmeth.1923 22388286 PMC3322381

[B51] Larson C. L. Wicht W. Jellison W. L. (1955). A new organism resembling *P. tularensis* isolated from water. *Publ. Health Rep.* 70 253–258. 10.2307/4589039 PMC2024510 14357545

[B52] Larsson P. Oyston P. C. Chain P. Chu M. C. Duffield M. Fuxelius H. H. (2005). The complete genome sequence of *Francisella tularensis*, the causative agent of tularemia. *Nat. Genet.* 37 153–159. 15640799 10.1038/ng1499

[B53] LeBel M. (1988). Ciprofloxacin: chemistry, mechanism of action, resistance, antimicrobial spectrum, pharmacokinetics, clinical trials, and adverse reactions. *Pharmacotherapy* 8 3–33. 10.1002/j.1875-9114.1988.tb04058.x 2836821

[B54] Loveless B. M. Yermakova A. Christensen D. R. Kondig J. P. Heine H. S. III Wasieloski L. P. (2010). Identification of ciprofloxacin resistance by SimpleProbe, high resolution melt and pyrosequencing nucleic acid analysis in biothreat agents: *Bacillus anthracis*, *Yersinia pestis* and *Francisella tularensis*. *Mol. Cell Probes* 24 154–160. 10.1016/j.mcp.2010.01.003 20100564

[B55] Luzzatto L. Apirion D. Schlessinger D. (1968). Mechanism of action of streptomycin in *E. coli*: interruption of the ribosome cycle at the initiation of protein synthesis. *Proc. Natl. Acad. Sci. U.S.A.* 60 873–880. 10.1073/pnas.60.3.873 4875806 PMC225133

[B56] Mahajan U. V. Gravgaard J. Turnbull M. Jacobs D. B. Mcnealy T. L. (2011). Larval exposure to *Francisella tularensis* LVS affects fitness of the mosquito *Culex quinquefasciatus*. *FEMS Microbiol. Ecol.* 78 520–530. 10.1111/j.1574-6941.2011.01182.x 22066999

[B57] Marcusson L. L. Frimodt-Møller N. Hughes D. (2009). Interplay in the selection of fluoroquinolone resistance and bacterial fitness. *PLoS Pathog.* 5:e1000541. 10.1371/journal.ppat.1000541 19662169 PMC2714960

[B58] Margolis J. J. El-Etr S. Joubert L. M. Moore E. Robison R. Rasley A. (2010). Contributions of *Francisella tularensis* subsp. novicida chitinases and Sec secretion system to biofilm formation on chitin. *Appl. Environ. Microbiol.* 76 596–608. 10.1128/aem.02037-09 19948864 PMC2805214

[B59] Martin M. (2011). Cutadapt removes adapter sequences from high-throughput sequencing reads. *EMBnet J.* 17:3.

[B60] Maruri F. Sterling T. R. Kaiga A. W. Blackman A. Van Der Heijden Y. F. Mayer C. (2012). A systematic review of gyrase mutations associated with fluoroquinolone-resistant *Mycobacterium tuberculosis* and a proposed gyrase numbering system. *J. Antimicrob. Chemother.* 67 819–831. 10.1093/jac/dkr566 22279180 PMC3299416

[B61] Matrat S. Aubry A. Mayer C. Jarlier V. Cambau E. (2008). Mutagenesis in the alpha3alpha4 GyrA helix and in the Toprim domain of GyrB refines the contribution of *Mycobacterium tuberculosis* DNA gyrase to intrinsic resistance to quinolones. *Antimicrob. Agents Chemother.* 52 2909–2914. 10.1128/aac.01380-07 18426901 PMC2493125

[B62] McKenna A. Hanna M. Banks E. Sivachenko A. Cibulskis K. Kernytsky A. (2010). The Genome Analysis Toolkit: a MapReduce framework for analyzing next-generation DNA sequencing data. *Genome Res.* 20 1297–1303. 10.1101/gr.107524.110 20644199 PMC2928508

[B63] Miskinyte M. Gordo I. (2013). Increased survival of antibiotic-resistant *Escherichia coli* inside macrophages. *Antimicrob. Agents Chemother.* 57 189–195. 10.1128/aac.01632-12 23089747 PMC3535928

[B64] Morner T. (1992). The ecology of tularaemia. *Rev. Sci. Tech.* 11 1123–1130. 10.20506/rst.11.4.657 1305858

[B65] Müller W. Hotzel H. Otto P. Karger A. Bettin B. Bocklisch H. (2013). German *Francisella tularensis* isolates from European brown hares (*Lepus europaeus*) reveal genetic and phenotypic diversity. *BMC Microbiol.* 13:61. 10.1186/1471-2180-13-61 23517149 PMC3663675

[B66] Nishimura K. Hosaka T. Tokuyama S. Okamoto S. Ochi K. (2007). Mutations in *rsmG*, encoding a 16S rRNA methyltransferase, result in low-level streptomycin resistance and antibiotic overproduction in *Streptomyces coelicolor* A3(2). *J. Bacteriol.* 189 3876–3883. 10.1128/jb.01776-06 17384192 PMC1913335

[B67] Oyston P. C. Quarry J. E. (2005). Tularemia vaccine: past, present and future. *Antonie Van Leeuwenhoek* 87 277–281. 10.1007/s10482-004-6251-7 15928980

[B68] Parker J. (2001). “Streptomycin,” in *Encyclopedia of Genetics*, eds Brenner S. Miller J. H. (New York, NY: Academic Press), 1890–1891.

[B69] Pechous R. D. Mccarthy T. R. Zahrt T. C. (2009). Working toward the future: insights into *Francisella tularensis* pathogenesis and vaccine development. *Microbiol. Mol. Biol. Rev.* 73 684–711. 10.1128/mmbr.00028-09 19946137 PMC2786580

[B70] Pérez N. Johnson R. Sen B. Ramakrishnan G. (2016). Two parallel pathways for ferric and ferrous iron acquisition support growth and virulence of the intracellular pathogen *Francisella tularensis* Schu S4. *MicrobiologyOpen* 5 453–468. 10.1002/mbo3.342 26918301 PMC4905997

[B71] Perez-Castrillon J. L. Bachiller-Luque P. Martin-Luquero M. Mena-Martin F. J. Herreros V. (2001). Tularemia epidemic in northwestern Spain: clinical description and therapeutic response. *Clin. Infect. Dis.* 33 573–576. 10.1086/322601 11462198

[B72] Pumbwe L. Piddock L. J. V. (2002). Identification and molecular characterisation of CmeB, a *Campylobacter jejuni* multidrug efflux pump. *FEMS Microbiol. Lett.* 206 185–189. 10.1111/j.1574-6968.2002.tb11007.x 11814661

[B73] Rohmer L. Brittnacher M. Svensson K. Buckley D. Haugen E. Zhou Y. (2006). Potential source of *Francisella tularensis* live vaccine strain attenuation determined by genome comparison. *Infect. Immun.* 74 6895–6906. 10.1128/iai.01006-06 17000723 PMC1698093

[B74] Saslaw S. Eigelsbach H. T. Prior J. A. Wilson H. E. Carhart S. (1961). Tularemia vaccine study. II. Respiratory challenge. *Arch. Intern. Med.* 107 702–714. 10.1001/archinte.1961.03620050068007 13746667

[B75] Schmieder R. Edwards R. (2011). Quality control and preprocessing of metagenomic datasets. *Bioinformatics* 27 863–864. 10.1093/bioinformatics/btr026 21278185 PMC3051327

[B76] Schuldiner S. (2018). The *Escherichia coli* effluxome. *Res. Microbiol.* 169 357–362. 10.1016/j.resmic.2018.02.006 29574104

[B77] Sharma D. Cukras A. R. Rogers E. J. Southworth D. R. Green R. (2007). Mutational analysis of S12 protein and implications for the accuracy of decoding by the ribosome. *J. Mol. Biol.* 374 1065–1076. 10.1016/j.jmb.2007.10.003 17967466 PMC2200631

[B78] Shirtliff M. E. Mader J. T. Camper A. K. (2002). Molecular interactions in biofilms. *Chem. Biol.* 9 859–871. 10.1016/s1074-5521(02)00198-9 12204685

[B79] Siebert C. Lindgren H. Ferré S. Villers C. Boisset S. Perard J. (2019). *Francisella tularensis*: FupA mutation contributes to fluoroquinolone resistance by increasing vesicle secretion and biofilm formation. *Emerg. Microbes Infect.* 8 808–822. 10.1080/22221751.2019.1615848 31164053 PMC6566608

[B80] Siebert C. Villers C. Pavlou G. Touquet B. Yakandawala N. Tardieux I. (2020). *Francisella novicida* and *F. philomiragia* biofilm features conditionning fitness in spring water and in presence of antibiotics. *PLoS One* 15:e0228591. 10.1371/journal.pone.0228591 32023304 PMC7001994

[B81] Springer B. Kidan Y. G. Prammananan T. Ellrott K. Böttger E. C. Sander P. (2001). Mechanisms of streptomycin resistance: selection of mutations in the 16S rRNA gene conferring resistance. *Antimicrob. Agents Chemother.* 45 2877–2884. 10.1128/aac.45.10.2877-2884.2001 11557484 PMC90746

[B82] Sreevatsan S. Pan X. Stockbauer K. E. Williams D. L. Kreiswirth B. N. Musser J. M. (1996). Characterization of *rpsL* and *rrs* mutations in streptomycin-resistant *Mycobacterium tuberculosis* isolates from diverse geographic localities. *Antimicrob. Agents Chemother.* 40 1024–1026. 10.1128/aac.40.4.1024 8849220 PMC163252

[B83] Sutera V. Levert M. Burmeister W. P. Schneider D. Maurin M. (2014). Evolution toward high-level fluoroquinolone resistance in *Francisella* species. *J. Antimicrob. Chemother.* 69 101–110. 10.1093/jac/dkt321 23963236

[B84] Toivonen J. M. Boocock M. R. Jacobs H. T. (1999). Modelling in *Escherichia coli* of mutations in mitoribosomal protein S12: novel mutant phenotypes of *rpsL*. *Mol. Microbiol.* 31 1735–1746. 10.1046/j.1365-2958.1999.01307.x 10209746

[B85] Tomaso H. Hotzel H. Otto P. Myrtennäs K. Forsman M. (2017). Antibiotic susceptibility *in vitro* of *Francisella tularensis* subsp. holarctica isolates from Germany. *J. Antimicrob. Chemother.* 72 2539–2543. 10.1093/jac/dkx182 28605439

[B86] van Hoek M. L. (2013). Biofilms: an advancement in our understanding of *Francisella* species. *Virulence* 4 833–846. 10.4161/viru.27023 24225421 PMC3925715

[B87] Vila J. Ruiz J. Goñi P. De Anta M. T. (1996). Detection of mutations in *parC* in quinolone-resistant clinical isolates of *Escherichia coli*. *Antimicrob. Agents Chemother.* 40 491–493. 10.1128/aac.40.2.491 8834907 PMC163143

[B88] Vila J. Soto S. M. (2012). Salicylate increases the expression of *marA* and reduces *in vitro* biofilm formation in uropathogenic *Escherichia coli* by decreasing type 1 fimbriae expression. *Virulence* 3 280–285. 10.4161/viru.19205 22546909 PMC3442840

[B89] Vuilleumier S. Yearsley J. M. Perrin N. (2008). The fixation of locally beneficial alleles in a metapopulation. *Genetics* 178:467. 10.1534/genetics.107.081166 18202388 PMC2206094

[B90] Wong A. Rodrigue N. Kassen R. (2012). Genomics of adaptation during experimental evolution of the opportunistic pathogen *Pseudomonas aeruginosa*. *PLoS Genet.* 8:e1002928. 10.1371/journal.pgen.1002928 23028345 PMC3441735

[B91] Yan J. Bassler B. L. (2019). Surviving as a community: antibiotic tolerance and persistence in bacterial biofilms. *Cell Host Microbe* 26 15–21. 10.1016/j.chom.2019.06.002 31295420 PMC6629468

[B92] Yoshida H. Bogaki M. Nakamura M. Nakamura S. (1990). Quinolone resistance-determining region in the DNA gyrase *gyrA* gene of *Escherichia coli*. *Antimicrob. Agents Chemother.* 34 1271–1272. 10.1128/aac.34.6.1271 2168148 PMC171799

[B93] Zogaj X. Wyatt G. C. Klose K. E. (2012). Cyclic di-GMP stimulates biofilm formation and inhibits virulence of *Francisella novicida*. *Infect. Immun.* 80 4239–4247. 10.1128/iai.00702-12 22988021 PMC3497427

