# Supplementary figures and images for: Evolution of Antibiotic Resistance in Surrogates of Francisella tularensis (LVS and Francisella novicida): Effects on Biofilm Formation and Fitness
Source: Front Microbiol. 2020 Oct 30;11:593542. doi: 10.3389/fmicb.2020.593542 (PMC7661474; doi:10.3389/fmicb.2020.593542)

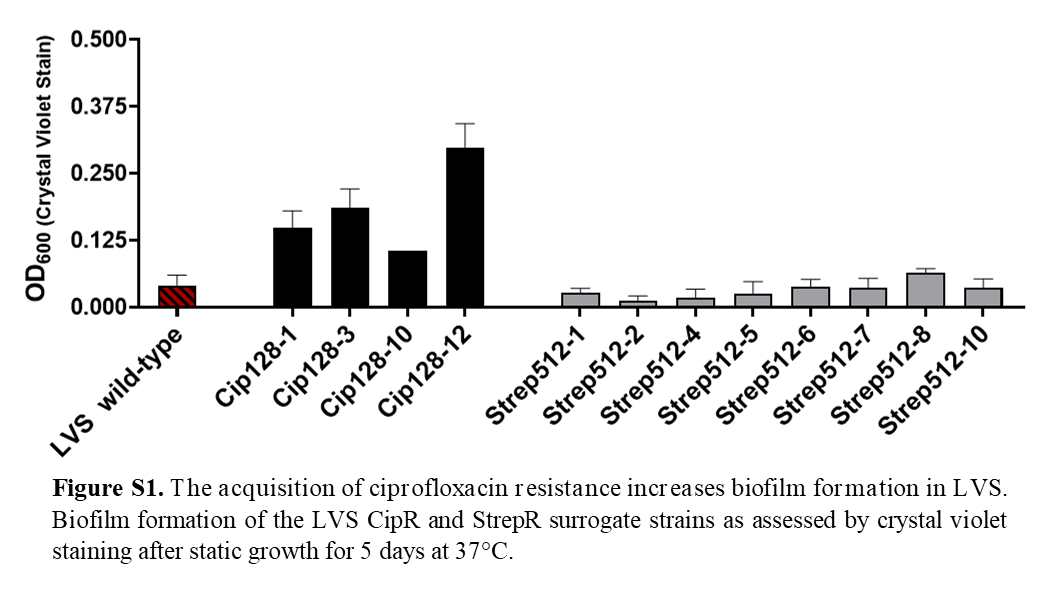

Supplement: Supplementary file 1 [file Image_1.tif]
