# Supplementary material for: Evolution of Antibiotic Resistance in Surrogates of Francisella tularensis (LVS and Francisella novicida): Effects on Biofilm Formation and Fitness
Source: Front Microbiol. 2020 Oct 30;11:593542. doi: 10.3389/fmicb.2020.593542 (PMC7661474; doi:10.3389/fmicb.2020.593542)
Supplement: Supplementary file 2 [file Table_1.docx]

**Table S1. Mutations identified among all CipR and StrepR *F. novicida* and LVS isolates***

| Locus | Type | Protein | Nucleotide change | Amino acid change | Severity |
| --- | --- | --- | --- | --- | --- |
| **Fn CipR** |  |  |  |  |  |
| AW25_RS00910 | frameshift_variant | thiopurine S-methyltransferase | 346_358delCCTAAGATAGCAA | Pro116fs | high |
| AW25_RS01955 | inframe_insertion | MexH family multidrug efflux RND transporter | 785_787dupATC | Asp262_Pro263insHis | moderate |
| AW25_RS02595 | missense_variant | *gyrA*, DNA Gyrase subunit A | 248C>T | Thr83Ile | moderate |
|  | missense_variant |  | 259G>T | Asp87Tyr | moderate |
| AW25_RS02890 | frameshift_variant | Epimerase | 59_60dupCG | Gly21fs | high |
| AW25_RS07640 | missense_variant | *parC*, DNA topoisomerase IV subunit A | 242G>A | Gly81Asp | moderate |
| AW25_RS06100-AW25_RS06105 | intergenic_region | N/A | 1278988C>T |  | modifier |
| **Fn StrepR** |  |  |  |  |  |
| AW25_RS08975 | missense_variant | 30S ribosomal protein S12 | 128A>G | Lys43Arg | moderate |
| **LVS CipR** |  |  |  |  |  |
| AW21_RS01670 | missense_variant | *gyrB*, DNA Gyrase subunit B | 1394C>A | Ser465Tyr | moderate |
| AW21_RS02275 | missense_variant | multidrug transporter AcrB; MMPL family transporter | 1958G>A | Arg653His | moderate |
| AW21_RS02545 | frameshift_variant | outer membrane protein assembly factor BamB | 870delT | Asp290fs | high |
| AW21_RS02555 | missense_variant | DNA topoisomerase IV subunit B | 1340G>T | Ser447Ile | moderate |
| AW21_RS03270 | missense_variant | Outer membrane efflux protein TolC | 1357C>A | Arg453Ser | moderate |
| AW21_RS03145-AW21_RS03150 | intergenic_region | N/A | 599763C>A |  | modifier |
| AW21_RS06025 | synonymous_variant | DUF3573 domain-containing protein FupA | 105G>C | Gly35Gly | low |
|  | frameshift_variant | DUF3573 domain-containing protein FupA | 107dupC | Leu37fs | high |
| AW21_RS06550 | missense_variant | DNA gyrase subunit A | 248C>T | Thr83Ile | moderate |
| AW21_RS06550 | frameshift_variant | DNA gyrase subunit A | 675delT | Gly225fs | High |
| AW21_RS06205 | missense_variant | transglycosylase SLT domain protein | 1868A>T | Lys623Ile | moderate |
| AW21_RS06850 | inframe_deletion | NAD-dependent epimerase/dehydratase family protein WbtC | 499_510delAAACTTGCAAAG | Lys167_Lys170del | moderate |
| AW21_RS09925 | stop_gained | lipopolysaccharide-assembly family protein LptE | 179T>G | Leu60* | high |
| AW21_RS10125 | frameshift_variant&stop_lost | IS630 family transposase | 502dupT | Ser168fs | high |
| **LVS StrepR** |  |  |  |  |  |
| AW21_RS00170 | missense_variant | MFS transporter | 53G>T | Trp18Leu | moderate |
| AW21_RS00955-AW21_RS10080 | intergenic_region | 10080- IS630 family transposase | 164135C>T |  | modifier |
| AW21_RS10125 | frameshift_variant&stop_lost | IS630 family transposase, pseudogene | 133dupT | Tyr45fs | high |
| AW21_RS01780 | missense_variant | IS5/IS1182 family transposase, pseudogene | 425T>C | Ile142Thr | moderate |
| AW21_RS10175 | synonymous_variant | IS630 family transposase, pseudogene | 162C>T | Ile54Ile | low |
| AW21_RS04075 | missense_variant | ribosomal RNA small subunit methyltransferase G (rsmG) | 10A>T | Met4Leu | moderate |
|  | frameshift_variant | ribosomal RNA small subunit methyltransferase G (rsmG) | 15delA | Asp6fs | high |
|  | synonymous_variant | ribosomal RNA small subunit methyltransferase G (rsmG) | 60T>C | Thr20Thr | low |
|  | missense_variant | ribosomal RNA small subunit methyltransferase G (rsmG) | 92T>C | Leu31Pro | moderate |
|  | frameshift_variant | ribosomal RNA small subunit methyltransferase G (rsmG) | 96_97insA | Leu33fs | high |
|  | missense_variant | ribosomal RNA small subunit methyltransferase G (rsmG) | 236G>T | Gly79Val | Moderate |
|  | stop_gained | ribosomal RNA small subunit methyltransferase G (rsmG) | 526G>T | Glu176* | High |
|  | missense_variant | ribosomal RNA small subunit methyltransferase G (rsmG) | 568C>T | Pro190Ser | Moderate |
|  | stop_gained | ribosomal RNA small subunit methyltransferase G (rsmG) | 583G>T | Glu195* | High |
| AW21_RS04995 | missense_variant | 30S ribosomal protein S12, rpsL | 263A>G | Lys88Arg | Moderate |
| AW21_RS06710 | frameshift_variant | two-component sensor histidine kinase | 457 A>T |  |  |
|  |  | two-component sensor histidine kinase | 458 G>C |  |  |
|  |  | two-component sensor histidine kinase | 459 T>C |  |  |
|  |  | two-component sensor histidine kinase | 460 T>C |  |  |
|  |  | two-component sensor histidine kinase | 462 del GAGGATC | 462fs |  |
|  |  | two-component sensor histidine kinase | 472 A>G |  |  |
|  |  | two-component sensor histidine kinase | 473 G>T |  |  |
|  |  | two-component sensor histidine kinase | 474 del C |  |  |
| AW21_RS06960 | missense_variant | recombination factor protein RarA, pseudogene | 719C>A | Pro240Gln | Moderate |
| AW21_RS07120 | stop_gained | aminotransferase, pseudogene | 620T>G | Leu207* | High |
| AW21_RS08665 | missense_variant | hypothetical protein | 725C>T | Ala242Val | moderate |
|  | missense_variant |  | 737C>G | Thr246Arg | moderate |
| AW21_RS09100 | missense_variant | NAD(P)/FAD-dependent oxidoreductase | 23G>T | Gly8Val | moderate |

*Summary of mutations identified from whole genome sequencing of LVS and *F. novicida* clones passaged upon ciprofloxacin and streptomycin. Upon sequencing of the final CipR and StrepR clones, we observed a diverse array of mutations, which ranged in severity from synonymous mutations (Low), missense variants or in-frame deletions (Moderate), and frameshift or nonsense mutations (High). Intergenic mutations were less common compared to mutations found within coding sequences, and were classified as Modifiers. Mutations were also classified as on-pathway, if they were known/ suspected to contribute directly to antibiotic resistance, or off-pathway, if they were not expected to contribute to antibiotic resistance.
