# Supplementary material for: Evolution of Antibiotic Resistance in Surrogates of Francisella tularensis (LVS and Francisella novicida): Effects on Biofilm Formation and Fitness
Source: Front Microbiol. 2020 Oct 30;11:593542. doi: 10.3389/fmicb.2020.593542 (PMC7661474; doi:10.3389/fmicb.2020.593542)
Supplement: Supplementary file 3 [file Table_2.docx]

**Table S2. Presence of mutations across the CipR and StrepR surrogate panel*.**

| Locus | Protein | Amino acid change | Surrogate strain | | | | | | | | | |
| --- | --- | --- | --- | --- | --- | --- | --- | --- | --- | --- | --- | --- |
| **Fn CipR** |  |  | Cip80-1 | Cip80-2 | Cip80-3 | Cip80-4 | Cip80-5 | Cip80-6 | Cip80-8 | Cip80-9 | Cip80-10 |  |
| AW25_RS00910 | thiopurine S-methyltransferase | Pro116fs | X | X | X | X | X | X | X | X | X |  |
| AW25_RS01955 | MexH family multidrug efflux RND transporter | Asp262_Pro263insHis | X | X | X | X | X | X | X | X | X |  |
| AW25_RS02595 | *gyrA*, DNA Gyrase subunit A | Thr83Ile | X | X | X | X | X | X | X | X | X |  |
|  |  | Asp87Tyr | X | X | X | X | X | X | X | X | X |  |
| AW25_RS02890 | SDR family oxidoreductase | Gly21fs | X | X | X | X | X | X | X | X | X |  |
| AW25_RS07640 | *parC*, DNA topoisomerase IV subunit A | Gly81Asp | X | X | X | X | X | X | X | X | X |  |
| AW25_RS06100-AW25_RS06105 | N/A |  |  |  |  |  |  |  |  |  | X |  |
| **Fn StrepR** |  |  | Strep512-1 | Strep512-2 | Strep512-3 | Strep512-4 | Strep512-5 | Strep512-6 | Strep512-7 | Strep512-8 | Strep512-9 | Strep512-10 |
| AW25_RS08975 | 30S ribosomal protein S12 | Lys43Arg | X | X | X | X | X | X | X | X | X | X |
| **LVS CipR** |  |  | Cip128-1 | Cip128-3 | Cip128-4 | Cip128-5 | Cip128-6 | Cip128-7 | Cip128-9 | Cip128-10 | Cip128-12 |  |
| AW21_RS01670 | *gyrB*, DNA Gyrase subunit B | Ser465Tyr | X | X | X | X | X | X | X | X | X |  |
| AW21_RS02275 | multidrug transporter AcrB; MMPL family transporter | Arg653His | X | X | X | X | X | X | X | X | X |  |
| AW21_RS02545 | outer membrane protein assembly factor BamB | Asp290fs | X | X | X | X | X | X | X | X | X |  |
| AW21_RS02555 | DNA topoisomerase IV subunit B | Ser447Ile | X | X | X | X | X | X | X | X | X |  |
| AW21_RS03270 | Outer membrane efflux protein TolC | Arg453Ser | X | X | X | X | X | X | X | X | X |  |
| AW21_RS03145-AW21_RS03150 | N/A |  | X | X | X | X | X | X | X | X | X |  |
| AW21_RS06025 | DUF3573 domain-containing protein FupA | Gly35Gly | X |  | X | X |  | X |  | X |  |  |
|  |  | Leu37fs | X | X | X | X | X | X | X | X | X |  |
| AW21_RS06550 | DNA gyrase subunit A | Thr83Ile | X | X | X | X | X | X | X | X | X |  |
| AW21_RS06205 | transglycosylase SLT domain protein | Lys623Ile |  |  | X | X |  | X |  | X |  |  |
| AW21_RS06850 | NAD-dependent epimerase/dehydratase family protein WbtC | Lys167_Lys170del | X | X | X | X | X | X | X | X | X |  |
| AW21_RS09925 | lipopolysaccharide-assembly family protein LptE | Leu60* |  | X |  |  | X |  | X |  |  |  |
| AW21_RS10125 | IS630 family transposase | Ser168fs |  |  | X | X | X |  | X |  |  |  |
| **LVS StrepR** |  |  | Strep512-1 | Strep512-2 | Strep512-3 | Strep512-4 | Strep512-5 | Strep512-6 | Strep512-7 | Strep512-8 | Strep512-9 | Strep512-10 |
| AW21_RS00170 | MFS transporter | Trp18Leu |  |  |  |  | X |  | X | X | X |  |
| AW21_RS00955-AW21_RS10080 | 10080- IS630 family transposase |  | X |  |  |  |  |  |  |  |  |  |
| AW21_RS10125 | IS630 family transposase, pseudogene | Tyr45fs | X |  | X | X |  |  | X | X | X |  |
| AW21_RS01780 | IS5/IS1182 family transposase, pseudogene | Ile142Thr |  |  |  |  |  | X |  |  |  |  |
| AW21_RS10175 | IS630 family transposase, pseudogene | Ile54Ile | X | X | X | X | X | X | X | X | X | X |
| AW21_RS04075 | ribosomal RNA small subunit methyltransferase G (rsmG) | Met4Leu | X |  |  |  |  |  |  |  |  |  |
|  |  | Asp6fs | X |  |  |  |  |  |  |  |  |  |
|  |  | Thr20Thr |  |  |  |  |  | X |  |  |  |  |
|  |  | Leu31Pro |  |  |  |  |  | X |  |  |  |  |
|  |  | Leu33fs |  |  |  |  |  | X |  |  |  |  |
|  |  | Gly79Val |  |  | X | X | X |  | X | X | X |  |
|  |  | Glu176* |  |  |  |  |  |  |  |  |  | X |
|  |  | Pro190Ser | X |  |  |  |  |  |  |  |  |  |
|  |  | Glu195* |  | X |  |  |  |  |  |  |  |  |
| AW21_RS04995 | 30S ribosomal protein S12, rpsL | Lys88Arg | X | X | X | X | X | X | X | X | X | X |
| AW21_RS06710 | *two-component sensor histidine kinase | fs at 462 | X |  |  |  |  |  |  |  |  |  |
|  |  |  | X |  |  |  |  |  |  |  |  |  |
|  |  |  | X |  |  |  |  |  |  |  |  |  |
|  |  |  | X |  |  |  |  |  |  |  |  |  |
|  |  |  | X |  |  |  |  |  |  |  |  |  |
|  |  |  | X |  |  |  |  |  |  |  |  |  |
|  |  |  | X |  |  |  |  |  |  |  |  |  |
|  |  |  | X |  |  |  |  |  |  |  |  |  |
| AW21_RS06960 | recombination factor protein RarA, pseudogene | Pro240Gln |  |  |  |  |  | X |  |  |  |  |
| AW21_RS07120 | aminotransferase, pseudogene | Leu207* |  |  |  | X |  |  |  |  |  |  |
| AW21_RS08665 | hypothetical protein | Ala242Val | X |  |  |  |  |  |  |  |  |  |
|  |  | Thr246Arg | X |  |  |  |  |  |  |  |  |  |
| AW21_RS09100 | NAD(P)/FAD-dependent oxidoreductase | Gly8Val |  |  |  |  |  |  |  |  |  | X |

*****All mutations identified in Fn and LVS upon passaging on ciprofloxacin or streptomycin are shown. An X indicates the presence of this mutation in the given strain.
